# CD34^+^ HSPCs-derived exosomes contain dynamic cargo and promote their migration through functional binding with the homing receptor E-selectin

**DOI:** 10.3389/fcell.2023.1149912

**Published:** 2023-04-25

**Authors:** Ioannis Isaioglou, Mansour M. Aldehaiman, Yanyan Li, Abdellatif Ait Lahcen, Sakandar Rauf, Asma S. Al-Amoodi, Umme Habiba, Abdullah Alghamdi, Shuho Nozue, Satoshi Habuchi, Khaled N. Salama, Jasmeen S. Merzaban

**Affiliations:** ^1^ Bioscience Program, Biological and Environmental Science and Engineering Division, King Abdullah University of Science and Technology (KAUST), Thuwal, Saudi Arabia; ^2^ Electrical and Computer Engineering Program, Computer, Electrical and Mathematical Science and Engineering Division, King Abdullah University of Science and Technology (KAUST), Thuwal, Saudi Arabia; ^3^ KAUST Smart-Health Initiative, King Abdullah University of Science and Technology, Thuwal, Saudi Arabia

**Keywords:** exosomes, CD34^+^ hematopoietic progenitor stem cells, proteomics, E-selectin, adhesion, migration, cargo alterations

## Abstract

Exosomes are tiny vesicles released by cells that carry communications to local and distant locations. Emerging research has revealed the role played by integrins found on the surface of exosomes in delivering information once they reach their destination. But until now, little has been known on the initial upstream steps of the migration process. Using biochemical and imaging approaches, we show here that exosomes isolated from both leukemic and healthy hematopoietic stem/progenitor cells can navigate their way from the cell of origin due to the presence of sialyl Lewis X modifications surface glycoproteins. This, in turn, allows binding to E-selectin at distant sites so the exosomes can deliver their messages. We show that when leukemic exosomes were injected into NSG mice, they traveled to the spleen and spine, sites typical of leukemic cell engraftment. This process, however, was inhibited in mice pre-treated with blocking E-selectin antibodies. Significantly, our proteomic analysis found that among the proteins contained within exosomes are signaling proteins, suggesting that exosomes are trying to deliver active cues to recipient cells that potentially alter their physiology. Intriguingly, the work outlined here also suggests that protein cargo can dynamically change upon exosome binding to receptors such as E-selectin, which thereby could alter the impact it has to regulate the physiology of the recipient cells. Furthermore, as an example of how miRNAs contained in exosomes can influence RNA expression in recipient cells, our analysis showed that miRNAs found in KG1a-derived exosomes target tumor suppressing proteins such as PTEN.

## Introduction

Exosomes are a subclass of membrane extracellular vehicles of endosomal origin (Ruivo et al., 2017; McAndrews and Kalluri, 2019) ranging in size from about 40 to 160 nm in diameter (Zhu et al., 2020) and carrying a diverse set of cargo, including mRNA, micro-RNA, long non-coding-RNA, soluble and transmembrane proteins, metabolites, lipids, and DNA (Balaj et al., 2011; Doyle and Wang, 2019; McAndrews and Kalluri, 2019; Patel et al., 2019). Exosomes have been isolated from many cell types, including stem cells and cancer cells (Ruivo et al., 2017; McAndrews and Kalluri, 2019; Nikfarjam et al., 2020; Zhu et al., 2020). It has been suggested that cancer cells of solid tumors use exosomes as communication vehicles to send cues to prepare the microenvironments of new locations where they want to spread (Mathivanan et al., 2010; Mathieu et al., 2019; McAndrews and Kalluri, 2019). Interestingly, leukemic cell derived exosomes can also cause alterations to their microenvironments (Kumar et al., 2016; Cunnane et al., 2018; Kumar et al., 2018).

Exosomes derived from Acute Myeloid Leukemia (AML) caused downregulation of the expression of stromal cell factor (SCF) and CXCL12 in stromal cells, both important for normal hematopoiesis which leads hematopoietic stem/progenitor cells (HSPCs) to leave the bone marrow (Huan et al., 2015; Yang et al., 2019). Furthermore, AML-derived exosomes can inhibit the cytolytic activity of natural killer cells and slow down their migration rate to subdue their anti-leukemia effects (Hong et al., 2017). Interestingly, AML-derived exosomes carry both myeloid-blast (i.e., CD33 and CD34) and leukemia-related (i.e., CD44 and CD123) markers (Szczepanski et al., 2011; Hong et al., 2014; Hong et al., 2017; Boyiadzis and Whiteside, 2018) suggesting that they mimic some of the cell surface markers present on their parent cells. Additionally, the exosomes ability to interact with different cell types likely emanates from the plethora of the ligands present on their surface.

CD34 and CD44 are well characterized E-selectin ligands (Dimitroff et al., 2001; AbuSamra et al., 2017). E-selectin is an adhesion molecule, constitutively expressed on the bone marrow endothelial cells and is responsible for the recruitment of HSPCs to the bone marrow (Schweitzer et al., 1996; Winkler et al., 2012). E-selectin interacts with its ligands, in a calcium-dependent glycosylation specific manner where the ligands present a posttranslational modification termed sialyl-Lewis X (sLe^x^) (McEver et al., 1995). We hypothesized that since hematopoietic (Merzaban et al., 2011; AbuSamra et al., 2017) and leukemic cells (Krause et al., 2014; Spertini et al., 2019) use E-selectin ligands to migrate to sites expressing selectins, exosomes derived from such cells would also bind and migrate to such sites. Indeed, we show that exosomes derived from a CD34^+^ progenitor AML cell line (KG-1a), as well as CD34^+^ cells isolated from healthy and AML donor bone marrow, contain E-selectin ligands and are functionally able to bind E-selectin in a Ca^2+^-dependent and sLe^x^ dependent manner. Furthermore, exosomes derived from a cell line that does not bind E-selectin—Namely the chronic myelogenous leukemia cell line, K562—also failed to bind E-selectin. Yet it could be coerced to bind through enforced formation of sLe^x^ following treatment with recombinant fucosyltransferase. Data presented here delve into understanding the mechanisms controlling exosome adhesion and migration, potentially revealing important aspects of the cancer metastatic cascade and, ultimately, presenting novel avenues for therapeutic intervention.

## Materials and methods

*Cell Culture:* The KG-1a cell line and the K562 cell line were purchased from the American Type Culture Collection (ATCC). KG-1a cells were cultured in Roswell Park Memorial Institute media (RPMI 1640, Thermo Fisher), and K562 cells were cultured in Iscove’s Modified Dulbecco’s Medium (IMDM, Thermo Fisher). Both culture media contained 10% Fetal Bovine Serum (FBS) (Corning) and 1% HyClone Penicillin-Streptomycin (Sigma-Aldrich). Cells were maintained in a humidified incubator at 37°C with a constant CO_2_ concentration of 5% v/v. The cell cultures were passaged frequently to maintain the cells at 0.8 × 10^6^ cells/ml.

Primary human AML CD34^+^ cells from Mobilized Peripheral Blood were purchased from AllCells, while Primary Human CD34^+^ cells from Mobilized Peripheral Blood were purchased from Lonza. Both cell types were cultured in StemSpan™ SFEM II (StemCell Technologies), containing 10% StemSpan™ CD34^+^ Expansion Supplement (StemCell Technologies). Cells were maintained in a humidified incubator at 37°C with a constant CO_2_ concentration of 5% v/v for 5 days.

*Exosomes Isolation:* Exosomes derived from cell lines were isolated using a serial centrifugation approach (Supplementary Figure S1) (Patel et al., 2019). Briefly, 35 million cells were maintained in media lacking FBS for 48 h prior to exosomes isolation. The cell culture was initially centrifuged for 10 minutes at 300 x g. The resulting supernatant was then centrifuged for 30 min at 2000 x g, and then the newly resulting supernatant was centrifuged for 30 min at 15,600 x g. Finally, the new supernatant was centrifuged for 120 min at 100,000 x g resulting in a pellet that contains the exosomes. Exosomes were then washed with 1x Phosphate-Buffered Saline (PBS, Sigma-Aldrich) and centrifuged again for 120 min at 100,000 x g. The resulting pellet was resuspended in 150 μl of PBS to proceed with downstream assays. The first two centrifugation steps took place using the Allegra X-12R Centrifuge (Beckman Coulter) and the remaining steps using the Optima L-90K Ultracentrifuge (Beckman Coulter).

Exosomes derived from primary AML HSPCs were isolated using a modified protocol. The cell culture was proceeded as described above until the end of the first two centrifugation steps. Due to the small volume of the culture, the resulting supernatant was spun down using the 5415 R Centrifuge (Eppendorf) for 30 min at 16,000 x g. The new resulted supernatant contained the exosomes, free of any larger particles and used for the downstream assays.

*Dynamic Light Scattering (DLS):* Exosomes’ size was calculated using the Dynamic Light Scattering (DLS) assay. The Zetasizer Nano ZS machine by Malvern Panalytical was used. The following parameters were used: Absorption = 0, Refractive index = 1.37, PBS viscosity = 0.8882, PBS refractive index = 1.33, Equilibration time = 10 s, Temperature = 25°C.

*Scanning Electronic Microscopy (SEM):* 5 μl of exosomes sample were placed on laser scribed graphene (LSG) coated with nanostructured gold material. These slides were then attached to a staple pin. The samples were dried at room temperature, and afterward, they were coated with a layer of 1 nm iridium. The samples were imaged using the Quanta SEM machine (FEI Company; KAUST Imaging and Characterization Core Lab) at 10 kV.

*Nanoparticle Tracking Analysis (NTA):* Exosomes were diluted at 1:10 using PBS and were quantified using the NanoSight LM20 by Malvern Panalytical. The following parameters were used: Brightness = 0, Gain = 1.00, Detection Threshold = 69, Temperature = 22°C, Viscosity = 0.95, Capture for 60 s with 30 frames per second.

*Exosome lysis:* Triton™ X-100 (Electrophoresis, Fisher BioReagents™) was added to the exosomes resuspended in PBS, with final concentration 1% (v/v) (Osteikoetxea et al., 2015). The mixture was vortexed for 30 s followed by rotation at 4°C for 1 h. In turn, the sample was placed in the sonicator (2510 Branson) for five cycles of 30 s on/off. Finally, the samples were centrifuged at 16,000 x g at 4°C for 10 minutes (5415 R, Eppendorf), and the resulting supernatant—containing the exosomal proteins—was collected.

*Protein immunoprecipitation:* Protein G Dynabeads (Thermo Fisher) were incubated with the respective antibody (Supplementary Table S1) (or the rE-selectin-IgG (Al-Amoodi et al., 2022)) for 1 hour, rotating at 4°C. After a washing step with PBS, the beads were incubated with the lysate of 100 million exosomes, overnight, rotating at 4°C. After three washes with the PBS, the beads were resuspended in buffer containing 50% NuPAGE^®^ LDS Sample Buffer (Thermo Fisher) and 50% PBS. The mixture was heated up at 90°C for 10 minutes and, in turn, was placed in a magnetic rack. The resulted supernatant was the immunoprecipitated product, and it was used for western blot.

Regarding the immunoprecipitation using the rE-selectin-IgG, the sample was split into two equal parts and to each one either calcium [2 mM] or EDTA [20 mM] was added. The calcium and the EDTA were also present at the respective concentrations for the washing steps.

*Western blot:* Exosomes resuspended in PBS were mixed with NuPAGE^®^ LDS Sample Buffer (Thermo Fisher) to a final concentration of 1x. 100 mM dithiothreitol (DTT) was added to the sample when reducing conditions were required. Samples were heated up to 90°C for 10 minutes while shaking at 300 rpm. Next, sonication (2510 Branson Sonicator) for 5 minutes was performed. Samples were then run on Criterion TGX Precast Protein Gels of 4%–20% polyacrylamide gels (BioRad) followed by transfer to PVDF membrane of 45 μm pore size (Merck) for western blot analysis as previously described (Aleisa et al., 2020). The transfer buffer contained 25 mM Tris-base, 192 mM glycine, and 20% (v/v) methanol, all dissolved in ddH_2_O. After the transfer, the membranes were washed with 1x Tris Buffered Saline with Tween 20 (TBST) (Cell Signaling) and then blocked for 8 hours with 5% (w/v) Bovine Serum Albumin (Sigma-Aldrich) dissolved in 1x TBST on a shaker at 4°C. In turn, membranes were washed once with 1x TBST and then were incubated overnight with shaking at 4°C, with the respective primary antibody (Supplementary Table S2). The membranes were then washed three times with 1x TBST for 5 minutes each and were incubated with the respective secondary antibody for 45 min with shaking at room temperature. After three washes, the membranes were incubated for 1 minute with ECL Prime western Blotting System (Merck) and were imaged using the ChemiDoc MP Imaging System (Bio-Rad).

*Mass Spectrometry:* Sample preparation: In-gel digestion approach was used for the proteomics analysis (Fischer and Kessler, 2015). Briefly, 500 million exosomes were prepared (see western blot method) and run on Criterion TGX Precast Protein Gels of 4%–20% polyacrylamide gel. The gel was stained with staining buffer (0.5 M citric acid, 5% (v/v) absolute ethanol and 0.01% (w/v) Coomassie Brilliant Blue G-250 (MP Biomedicals)) and destained with destaining buffer (ddH_2_O 50% (v/v), methanol 40% (v/v) and acetic acid 10% (v/v)). The parts of the gel with protein sample were cut and segmented into smaller pieces and finally placed in tubes with destaining solution (200 mM Triethylammonium Bicarbonate (TEAB) (Thermo Fisher), 40% acetonitrile (ACN)), incubated at 37°C with shaking until there was no color observed in the gel pieces. Next, the supernatant was removed, and the gel pieces were placed in the Concentrator Plus (Eppendorf) until they were dry. Next, gel pieces were covered with 100 mM TEAB, and 0.4 μg of Sequencing Grade Modified Trypsin (Promega) was added. The digestion took place overnight at 37°C. The supernatant with the digested peptides was next transferred to a new tube to perform the desalting steps using C18 pipette tips (Agilent). Finally, the peptides were resuspended in 3% (v/v) ACN.

*Mass spectrometry analysis:* The Mass spectrometry analysis was performed at the KAUST Bioscience Core Lab as previously described (Liu et al., 2019). Briefly, the digested peptides were measured on a Q-Exactive HF mass spectrometer (Thermo Fisher Scientific) coupled with an UltiMate™ 3000 UHPLC (Thermo Fisher Scientific). For the healthy HSPCs-derived exosomes timsTOF Pro (Brucker) was used to increase sensitivity. The raw data were converted to Mascot generic format files and aligned to the Uniprot Human database. *In silico* analysis was performed using the DAVID online tool (Huang et al., 2009a; Huang et al., 2009b), and the illustrations were created *via* the KEGG Mapper online tool (Kanehisa and Goto, 2000; Kanehisa, 2019; Kanehisa et al., 2021).

*E-selectin constructs:* The recombinant E-selectin constructs used in this study were developed in a previous publication in our group (Aleisa et al., 2020). We used a recombinant E-selectin construct (“rE-selectin”), which corresponds to the native E-selectin, consisting of the extracellular part of E-selectin containing: A lectin domain, an EGF domain, and six Short Consensus Repeat (SCR) domains. We also used a recombinant soluble E-selectin construct (“rE-selectin-IgG”), which consists of two rE-selectin constructs linked together by the human Fc (IgG) region. Both constructs have at their C-terminal end His-tag and Strep-tag domains to facilitate downstream applications.

*Fucosyltransferase VI treatment:* K562-derived exosomes were isolated and treated with the recombinant human fucosyltransferase VI (rhFTVI) enzyme that was produced in our laboratory (Al-Amoodi et al., 2020). The treatment was performed in a 96-well plate in rhFTVI using 100 μL of 2X reaction buffer [25 mM HEPES (pH 7.5) (Gibco Invitrogen), 0.1% human serum albumin (Sigma-Aldrich), 0.5 mM GDP-fucose (Sigma), and 5 mM MnCl_2_], 100 μl of PBS containing 50 × 10^6^ exosomes, and approximately 1 μg of purified rhFTVI enzyme. Exosomes were incubated at 37°C for 40 min. Buffer-only control, without the rhFTVI enzyme, was used as a negative control (Mock). After the treatment, the exosomes were cleaned using the Exosome Spin Columns (Thermo Fisher) and then processed for downstream experiments.

*Exosomes Pulldown:* The rE-selectin-IgG was incubated with Protein G Dynabeads for 1 h while rotating at 4°C. Following a washing step, the Dynabeads coupled with rE-selectin-IgG were resuspended in PBS containing either 2 mM of Ca^2+^ or 20 mM of EDTA (Figure 3A). The sample that had Dynabeads without E-selectin was also resuspended in PBS containing 2 mM Ca^2+^. In each sample, about 4.5 × 10^6^ KG1a-exosomes were added and were incubated overnight, rotating at 4°C. Next, three washes using PBS with the respective Ca^2+^ or EDTA concentration took place, and finally, the E-selectin cargo was eluted. For the elution, 8M urea was used—Enough to cover all the beads- and a 5 min incubation at room temperature took place. Afterward, NuPAGE^®^ LDS Sample Buffer was added in equal volume to the urea, and was allowed to incubate for another 5 minutes at room temperature. In turn, the tubes were placed in a magnetic rack where the beads were separated from the supernatant. Then, the supernatant was removed to another tube and placed in a sonicator (2510 Branson) for 5 m. Finally, the samples were heated up for 10 minutes at 85°C and were run for western blot analysis.

FTVI-treated and buffer-treated K562-derived exosomes were incubated with Dynabeads coupled with rE-selectin-IgG in the presence of 2 mM Ca^2+^. After overnight incubation, the beads were washed three times with PBS containing 2 mM Ca^2+^ prior to elution using 50 mM EDTA for 5 min (incubated at room temperature). The downstream processing was the same as for the KG1a-derived exosomes. The methodology that was used for the pulldown of the exosomes derived from the primary cells, was the same as the one for the KG1a-derived exosomes.

*Exosomes staining*: Exosomes were stained with the Vybrant DiD Cell-Labeling Solution by Thermo Fisher. DiD was added to exosomes (−10^7^) diluted in PBS, so its final concentration is 2 μΜ. After incubating for 1 h at 37°C, the exosomes were cleaned using the Exosome Spin Columns (Thermo Fisher). For the *in vivo* experiments, exosomes were stained with the VivoTrack 680 by PerkinElmer. Vivo Track 680 was added to 2 × 10^7^ exosomes to a final concentration of 33 mΜ. After incubation for 15 min at 37°C, exosomes were cleaned using the Exosome Spin Columns (Thermo Fisher).

*Fluorescence imaging of exosomes:* To image exosomes, a wide field illumination fluorescence microscope mounted on with an inverted IX71 optical microscope platform (Olympus) was used (Alghamdi et al., 2023). A 638-nm (60 mW; MLD, Cobolt) continuous wave (CW) laser was introduced through a focusing lane lens (f = 300 mm; Thorlabs) to focus the laser beams at a back focal plane of ×100 objective lens [NA = 1.49; UAPON 100XOTIRF, Olympus]. An illumination excitation power of 14.60.35 mμW cm^-2^ was used. Fluorescence from the sample was detected by an iXon3 897 EMCCD EMCCD camera (Andor Technology) after passing through a dichroic mirror (FF660-Di02-25×36, Semrock) and an emission bandpass filter (F01-676/29LD01-640/8, Semrock) and a dichroic mirror (FF660-Di02-25 × 36, Semrock). This configuration separates the illumination excitation light from the sample fluorescence signal, allowing the sample fluorescence to be captured by the same objective. Images were recorded A 125 × 125-pixel region of the EMCCD camera with was used to record the sample fluorescence at a 30 m exposure time with EM gain of 300.

Commercially available recombinant human E-selectin (rhE-selectin) (1 μg/ml) (Sino Biological) was deposited into a µ-Slide VI 0.1 uncoated microfluidics chamber (channel width, 1 mm; channel height, 0.1 mm; ibidi GmbH) overnight at 4°C. The chamber was washed using PBS. Exosomes stained with DiD were resuspended in either 2 mM of Ca^+2^ or 20 mM of EDTA in PBS, introduced in the chamber *via* negative pressure (Figure 4A) and then incubated for 30 min at room temperature. The chamber was washed four times using PBS and then imaged. Exosomes were visualized using an Olympus IX71 inverted optical microscope outfitted with a UAPON 100XOTIRF high numerical aperture (NA) objective (Olympus). Transmitted optical microscopy images were captured using an iXon3 897 EMCCD camera (Andor Technology) through the Andor iQ3 software.

*Live fluorescence capturing of exosomes:* For real-time exosome capturing (Alghamdi et al., 2023), male Luer connectors (ibidi GmbH) were connected to the inlet and outlet of the chamber to allow connection with 0.8-mm silicone tubing (ibidi GmbH). The silicone tubing connected to the inlet was placed in a PBS rolling buffer made with 2 mM of Ca^+2^. The silicone tubing connected to the outlet was joined to a syringe pump (PHD ULTRA, Harvard Apparatus) by attaching a female Luer Lock connector (ibidi GmbH). Before perfusion of exosomes, the rolling buffer was flowed into the chamber to equilibrate the flow in the chamber. 10^7^of DiD-stained exosomes were resuspended in 200 μl of the rolling buffer then perfused into the rhE-selectin (1 μg/ml) deposited chamber at a flow rate beginning at 100 μl min^−1^. Once the exosomes were captured, the flow rate was maintained at 100 μl min^−1^ for 20 s and then systematically increased up to 2000 μl min^−1^ in the following order: 100, 200, 500, 1000, 2000 μl min^−1^ for 15–20 s at each step. The experimental setup could capture 14 frames per second.

*Microscale Thermophoresis (MST):* Molarity calculation- To perform MST analysis, the molarity of the two interacting parts is needed. The challenge was that the exosomes do not have any standard molecular weight, so we devised an alternative to calculate the molarity of the exosomes. By quantifying the exosomes as particles per mL using the Nanosight NTA machine, and by applying the Avogadro’s number, we were able to determine the molarity. Another challenge to our experiment was that the exosomes that we could isolate were about 10^8^–10^9^ particles per mL, equivalent to a molarity of 20–40 nM. Note that in MST, the non-labeled molecules should have a significantly higher concentration than the labeled molecules.

Sample preparation- The rE-selectin was labeled using the Monolith NT His-Tag Labeling Kit RED-tris-NTA (NanoTemper Technologies). To assess binding detection (*Binding Check*), the final concentration of the rE-selectin was 1 nM, and the final concentration of the exosomes was 11 nM. The mixture contained 0.05% Tween 20 and 2 mM Ca^+2^. After mixing, the samples were centrifuged for 10 min at 10,000 rpm and finally loaded onto Premium Capillaries (NanoTemper Technologies). The samples were run on the Monolith NT.115Pico (NanoTemper Technologies). For the binding affinity assay (*Binding Affinity*), the final concentration of the E-selectin was 0.25 nM. The first sample had an exosomes concentration of 11 nM, and every following sample had 50% of the exosome’s concentration of the previous one (i.e., the second sample had an exosomes concertation of 6.5 nM). Similar to the binding detection assay, samples contained 0.05% Tween 20 and 2 mM Ca^2+^ and were processed in a manner resembling the binding detection assay.

*E-selectin treatment:* After isolation, 200 million KG1a-derived exosomes were resuspended into 200 μl of PBS in the presence of 2 mM calcium. The mixture was spitted into two tubes and in one of them 2.5 μg of rE-selectin was added. The samples were incubated for 1 h at 37°C followed by addition of 40 μl of LDS to stop the reaction. The samples were heated up for lysis at 90°C for 10 minutes and then proceed for western blot.

*miRNA target analysis:* The miRNAs that existed in KG1a-derived exosomes were derived from Xu et al. (2020). The top 50 miRNAs according to their transcripts per million values were used and analyzed by using the MIENTURNET (Licursi et al., 2019) and the DIANA mirPath v.3 (Vlachos et al., 2015) online tools. The data presented here have an adjusted *p*-value (FDR) < 0.05 for the target-miRNA interaction.

*E-selectin blocker antibody purification:* H18/7 cell line was purchased from American Type Culture Collection (ATCC). Cells were cultured in Roswell Park Memorial Institute media (RPMI 1640, Thermo Fisher) contained 10% Fetal Bovine Serum (FBS) (Corning), 1% HyClone Penicillin-Streptomycin (Sigma-Aldrich) and 0.05% β-mercaptoethanol (Thermo Fisher). Cells were maintained in a humidified incubator at 37°C with a constant CO_2_ concentration of 5% v/v. Fresh media was added daily to the cell culture to maintain the cells at 0.5 × 10^6^ cells/mL. Once the total volume of the culture was 1 L, cells were harvested by centrifugation at 5,500 x g for 10 minutes twice and filtered to separate the supernatant from the cell pellet. Supernatant was loaded onto HiTrap HP protein A 1 ml column (GE Healthcare) using Fast Protein Liquid Chromatography (FPLC) equilibrated with a buffer containing: 10 mM Sodium phosphate and 150 mM NaCl (pH 7). Finally, bound protein was eluted with ten times the column volume using a buffer of 100 mM Citric acid (pH 3). Eluents were diluted and neutralized with a neutralization buffer of 1 M Tris (pH 9) and the protein was dialyzed overnight in PBS buffer.

*In vivo:* Non-obese diabetic (NOD) SCID Gamma (NSG) mice were purchased from Charles River company (Lodi, Italy) and maintained in the KAUST Animal Research Core Lab (ARCL) facility. NSG mice were randomly assigned to three groups: 1) Control group, 2) Exosomes group and 3) Exosomes + anti-E-selectin antibody group. The control group 1) Were injected intravenously 4) Into the tail vein with PBS buffer containing 33 mM of VivoTrack 680 but no exosomes (n = 3). Group 2) Were comprised of mice that received 300 × 10^6^ VivoTrack680-prestained KG1a-derived exosomes (n = 4) IV. While group 3) Were comprised of mice that were pretreated with 120 μg of E-selectin blocking antibody (H18/7). Three hours after the treatment, 300 × 10^6^ VivoTrack680-prestained KG1a-derived exosomes were intravenously delivered into the pretreated mice (n = 4). All samples were passed through an Exosome Spin Column (Thermo Fisher) to remove excess VivoTrack 680 prior to IV injection.

Exosome distribution in mice was imaged at 2, 6, 12, 24, and 48 h using the IVIS Spectrum (PerkinElmer Inc., MA, United States). After the last image, mice were euthanized, and organs (spleen, liver, spine, femur, and tibia) were harvested for *ex-vivo* imaging using the IVIS Spectrum. All images were acquired using a CCD camera with the following parameters: binning = medium; f/stop 2. Filter sets were fixed at 675 nm (excitation) and 720 nm (emission). Fluorescence intensity was measured and analyzed by the Living Image software (Caliper Life Sciences, MA, United States). The distribution of VivoTrack 680 in the whole body, spleen, kidney, spine, and hind legs was quantified by average radiant efficiency ([p/s/cm^2^/sr]/[μW/cm^2^]).

*Statistical analysis:* Results are represented as mean ± Standard Deviation. Statistical analysis was performed using the GraphPad Prism 9 software. The differences observed between different groups were compared, and statistical analysis was analyzed using a student’s t-test, and values with *p* < 0.05 were considered statistically significant.

## Results

### The proteome of HSPCs-derived exosomes is enriched with proteins related to cell adhesion and migration

**FIGURE 1:**
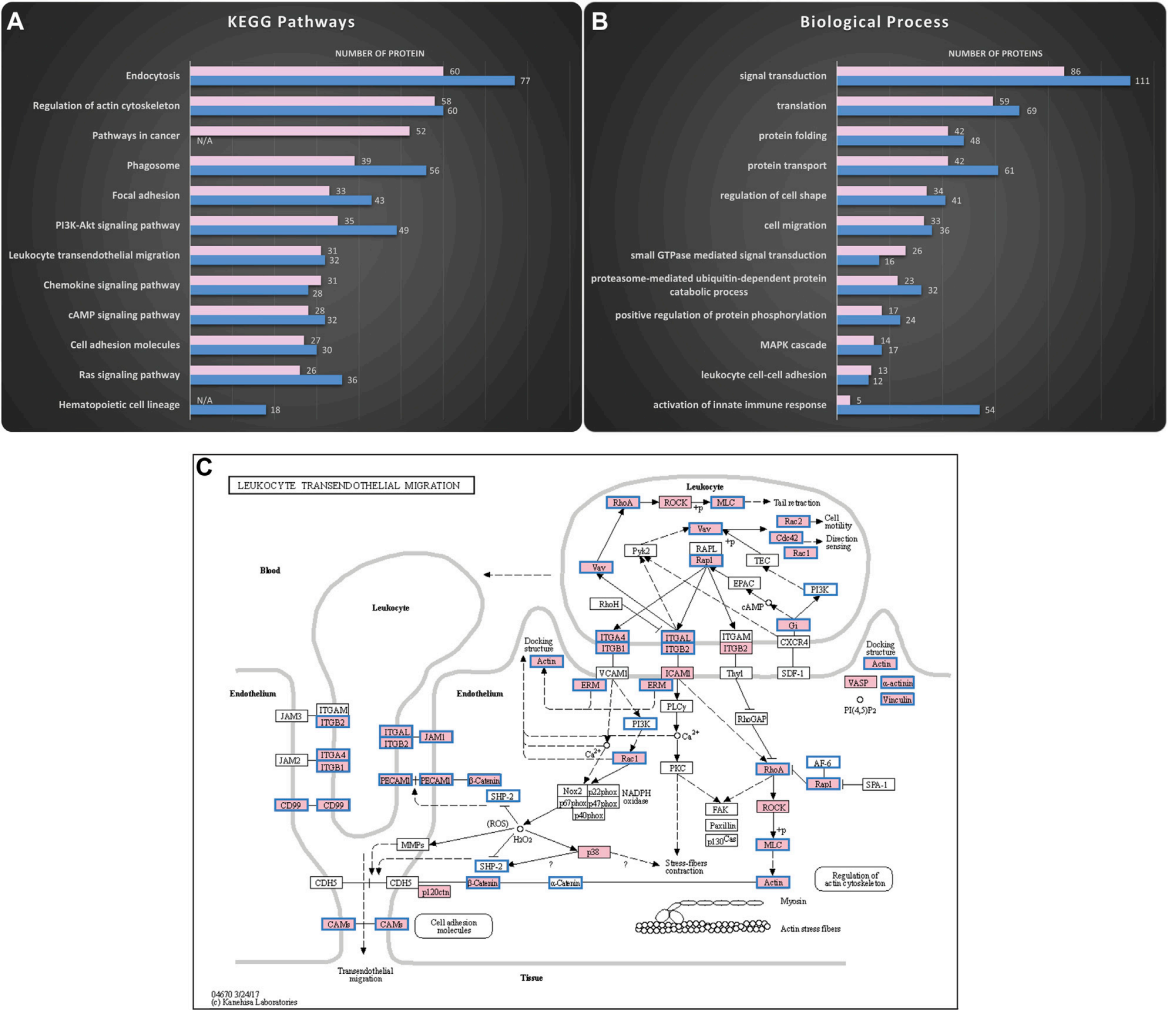
Mass spectrometry analysis reveals HSPCs-derived exosomes are enriched in proteins involved in adhesion and migration. Proteomics analysis of the cargo was performed to uncover putative functions of the isolated exosomes. The KEGG pathways and the Biological Processes from Gene Ontology analysis that were enriched in the KG1a (pink) and healthy HSPCs (blue)-derived exosomes were identified using the DAVID online tool. **(A)** Top hits of the KEGG pathways identified included pathways related to vehicle-trafficking and adhesion-migration functions. Furthermore, there was enrichment in signaling networks, highly related to the regulation of migration. The complete list of the identified KEGG pathways can be found in “HSPCs-derived exosomes proteome analysis.” Datasheet 2. **(B)** Top hits of Biological Processes identified included processes related to cell adhesion and signaling cascades, confirming the KEGG pathways. The complete list of identified Biological Processes can be found in “HSPCs-derive exosomes proteome analysis.” Datasheet 2. **(C)** KEGG map revealed proteins in KG1a (pink) and healthy HSPCs (blue frame)-derived exosomes related to Leukocyte Transendothelial Migration. Among the identified proteins, there were integrins (e.g., integrin-alpha), cytoskeleton-related proteins (e.g., actin, ezrin), and chemokines (e.g., IL-32).

Exosomes were isolated using serial centrifugation steps (Supplementary Figure S1). Standard quality control experiments (Lotvall et al., 2014) including DLS, SEM and western blot confirmed that the isolated particles were indeed enriched for exosomes (Supplementary Figure S2). Thereafter, mass spectrometric analysis was used to uncover the proteome cargo of the KG1a and healthy primary CD34^+^ HSPCs-derived exosomes (Figure 1). In each sample, about 2000 proteins were uncovered, including proteins typically found in exosomes such as CD63 and CD81, the programmed cell death 6- interacting protein (e.g., Alix), and the heat shock proteins 70 and 90 (HSP 70 & HSP 90) (Supplementary Figure S3) (Borges et al., 2013). *In silico* analysis was performed using the DAVID online tool (Huang et al., 2009a; Huang et al., 2009b), and the illustrations were created *via* the KEGG Mapper online tool (Kanehisa and Goto, 2000; Kanehisa, 2019; Kanehisa et al., 2021). As illustrated in Figure 1A, many of the proteins found in exosomes were related to vehicle-trafficking pathways such as endocytosis and phagosomes consistent with exosome’s biogenesis origin. Surprisingly, a considerable number of pathways were related to adhesion and migration functions such as focal adhesion and leukocyte transendothelial migration. Exosomes also carried proteins involved in signaling pathways such as Rap1, Ras, and chemokine signaling pathways. Interestingly, these signaling pathways are major regulators of cell adhesion and migration processes (van Buul and Hordijk, 2004; Castellano et al., 2016; Zhang et al., 2017). In addition, gene ontology analysis of the HSPCs-derived exosomal proteome revealed enrichment in biological processes highly related to the KEGG pathways mentioned above. As shown in Figure 1B, most of the biological processes were related to cell adhesion-migration (i.e., cell migration, regulation of cell shape, leukocyte cell-cell adhesion) or to signaling (i.e., signal transduction, small GTPase signaling, MAPK cascade), confirming the findings from the KEGG analysis. Therefore, the proteomics data analysis indicated a putative correlation of HSPCs-derived exosomes to adhesion and migration phenomena. Furthermore, the mass spectrometry data identified an abundance of proteins involved in the multistep paradigm of cell migration, such as selectin ligands, integrins, and chemokines. Moreover, E-selectin ligands such as CD34 (AbuSamra et al., 2017), CD44 (Dimitroff et al., 2001; Merzaban et al., 2011; AbuSamra et al., 2015; Ali et al., 2017; AbuZineh et al., 2018; Al Alwan et al., 2021), CD43 (Matsumoto et al., 2005) and PSGL-1 (Katayama et al., 2003; AbuSamra and Merzaban, 2015; Ali et al., 2017) were identified in the exosomes. Since the data suggest that these ligands are involved in cellular rolling and migration, we chose to focus on which of the identified exosomal proteins were part of the two pathways related to the multistep paradigm of cell migration and adhesion using the KEGG Mapper online tools: The “Leukocyte Transendothelial Migration” pathway (Figure 1C) and the “Cell Adhesion Molecules” pathway (Supplementary Figure S4).

### Hematopoietic cell-derived exosomes display E-selectin ligands

**FIGURE 2:**
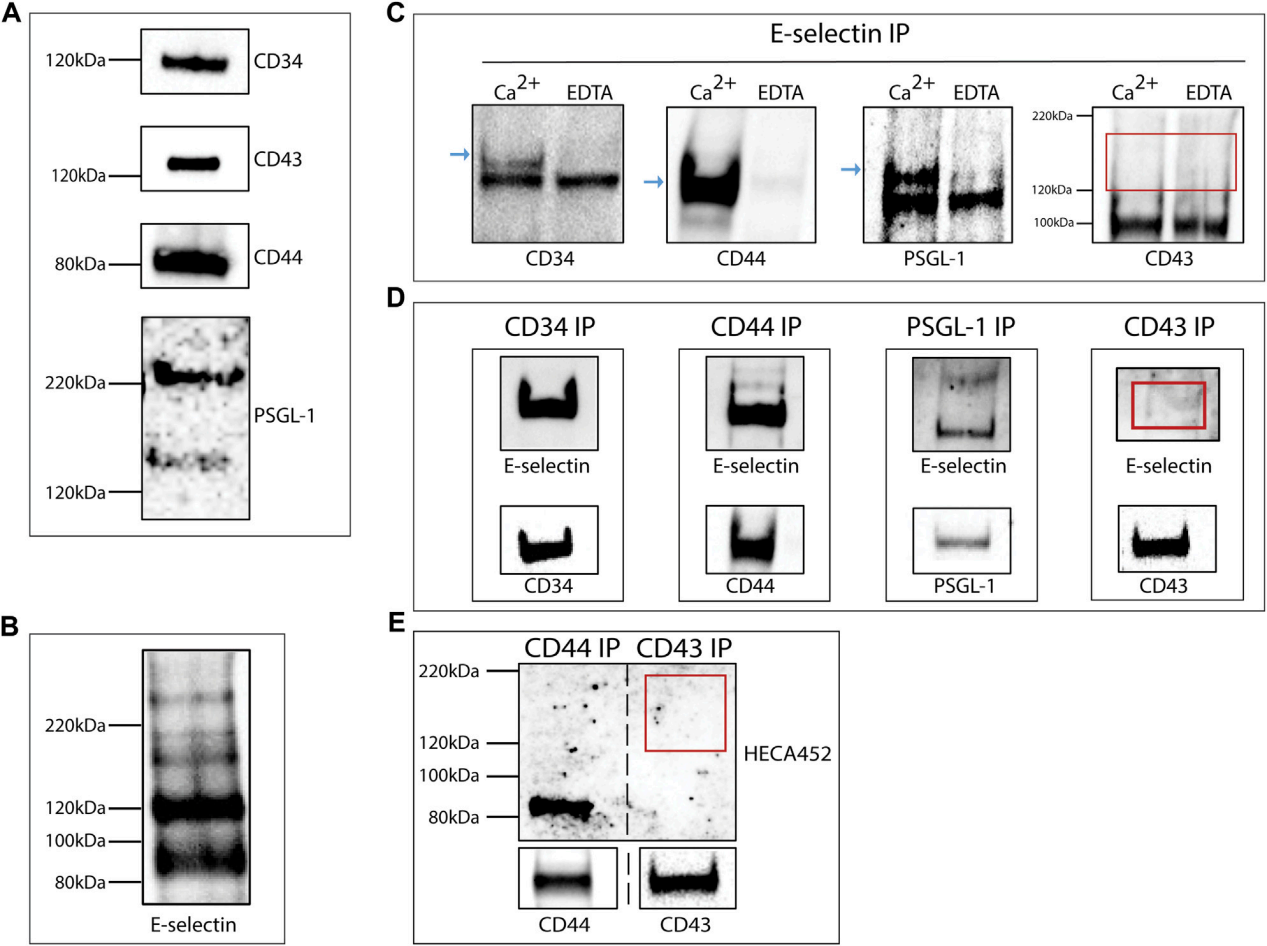
KG1a-derived exosomes express E-selectin ligands. **(A)** Western blot analysis of lysates of KG1a-derived exosomes revealed the presence of CD34, CD43, CD44, and PSGL-1 at the expected molecular weights. For PSGL-1, two bands were detected as expected (Snapp et al., 1998), a lower band corresponding to the monomeric form and a higher band corresponding to the dimeric form. **(B)** Western blot analysis of KG1a-derived exosome lysates stained with rE-selectin-IgG revealed several potential E-selectin ligands. **(C)** rE-selectin-IgG was used to immunoprecipitate (IP) proteins from lysates of KG1a-derived exosomes either in the presence of Ca^2+^ (2 mM) or EDTA (20 mM). Western blot analysis revealed the presence of CD34, CD44, and PSGL-1 (blue arrows) in samples where Ca^2+^ was added but not in samples containing EDTA confirming the selective, calcium-dependent binding of E-selectin to its ligands. In western blots for CD43, no band was observed at the expected MW (see red rectangle). **(D)** CD44, CD34, PSGL-1, and CD43 were immunoprecipitated (IP) from the lysates of KG1a-derived exosomes using antibodies against each potential E-selectin ligand. The IP product was blotted against the rE-selectin-IgG (*upper*) and each respective ligand (*lower*, positive control). A clear band corresponding to CD43 was not detected (see red rectangle). Note that at in the *upper* blots, double the amount of sample was loaded compared to the *lower* blots in order to establish that although a substantial amount of CD43 protein is present (*lower*), the lack of E-selectin binding (*upper*) is likely a result of the lack of proper glycosylation. **(E)** CD44 and CD43 were Immunoprecipitated (IP) from lysates of KG1a-derived exosomes using antibodies against each ligand. The IP product was blotted for sLe^x/a^ using the HECA-452 antibody (*upper*) and each specific ligand (*lower*). No band corresponding to CD43 was detected with HECA-452 (see red rectangle) even though CD43 was present (see *lower* CD43 blot) suggesting that this ligand does not express sLe^x/a^ structures. Results shown are representative of n = 3 independent experiments.

Western blot analysis of total lysates from the KG1a-derived exosomes confirmed the presence of E-selectin ligands: CD34, CD43, CD44, and PSGL-1 as suggested by the proteomics analysis (Figure 2A). The expression of these ligands alone does not dictate E-selectin binding since ligands need to be decorated with sialyl Lewis X (sLe^x^) glycans to be able to bind E-selectin. In order to determine if exosomes contained ligands that could bind E-selectin, a western blot analysis was performed on lysates of KG1a-derived exosomes blotted with the recombinant E-selectin IgG chimeric protein (rE-selectin-IgG). As shown in Figure 2B, several bands corresponding to different E-selectin ligands were detected.

To further investigate the interaction of E-selectin with the proposed exosomal ligands, immunoprecipitation on the total exosomal lysate was performed using the rE-selectin-IgG construct. To confirm the calcium-dependent binding of E-selectin to its ligands, immunoprecipitation was performed in the presence of Ca^2+^ or EDTA. As shown in Figure 2C, the immunoprecipitation products were blotted against the respective E-selectin ligands. CD34, CD44, and PSGL-1 blots showed a clear signal in the sample immunoprecipitated in the presence of Ca^2+^ but not in the presence of EDTA. Surprisingly, the blot of CD43 did not show any signal in neither Ca^2+^ nor EDTA immunoprecipitated samples. To further confirm our results, a complementary approach was used where each E-selectin ligand was immunoprecipitated (Figure 2D, *lower* blots) and subsequently blotted with rE-selectin-IgG (Figure 2D, *upper* blots). As illustrated in Figure 2D (*upper*), CD34, CD44, and PSGL-1 immunoprecipitates showed a well-defined band at the respective molecular weights. However, CD43 immunoprecipitates did not show any clear band at the molecular weight corresponding to CD43, in agreement with the previous results (Figure 2C).

E-selectin binds specialized carbohydrate determinants, comprised of sialofucosylations containing an α (McAndrews and Kalluri, 2019; Zhu et al., 2020)-linked sialic acid substitution on galactose, and an α(1,3)-linked fucose modification on N-acetylglucosamine, prototypically displayed as the terminal tetrasaccharide, sLe^x^. To assess whether or not the CD43 glycoprotein is decorated with sLe^x^ capped glycans (Nelson et al., 1993; Merzaban et al., 2011; AbuSamra et al., 2015; AbuSamra et al., 2017), western blots of CD43 immunoprecipitates were prepared and blotted with the anti-sLe^x/a^ antibody, HECA-452. The successful immunoprecipitation of CD43 is shown in Figure 2E, (*lower*). CD44 immunoprecipitates were run in parallel as a positive control (Dimitroff et al., 2001; Sackstein et al., 2008; AbuSamra et al., 2015). As illustrated in Figure 2E (*upper*), the sample corresponding to CD44 immunoprecipitates presented a band at the respective molecular weight of CD44 while CD43 immunoprecipitates did not. These findings suggest that although CD43 is present in the KG1a-derived exosomes, it is not decorated with the sLe^x^ glycan epitopes that are necessary for binding to E-selectin.

To further investigate the impact of sLe^x^ formation on the ability of exosomes to interact with E-selectin, exosomes were isolated from K562 (Al-Amoodi et al., 2020) cell line (Supplementary Figure S5A). K562 cells are not able to bind E-selectin, because their ligands are missing the necessary glycosylation. Using recombinant fucosyltransferase VI (FTVI), an enzyme, that is, not expressed in K562 cells but can aid in the creation of sLe^x^, E-selectin binding can be achieved (Al-Amoodi et al., 2020). Like the cells they originate from, the E-selectin ligands, CD44, CD43 and PSGL-1 were created on K562-derived exosomes (Supplementary Figure S5B). Following treatment with FTVI, lysates of K562-derived exosomes (FTVI) bound E-selectin as determined by western blot while lysates from buffer-treated K562-derived exosomes (Mock) did not (Supplementary Figure S5C). When rE-selectin-IgG was used to immunoprecipitated potential E-selectin ligands from lysates of either FTVI-treated or buffer-treated K562-dervied exosomes and subsequently analyzed by western blot for CD43, CD44 and PSGL-1, ligands were only found in lysates prepared from FTVI-treated exosomes (Supplementary Figure S5D). These data illustrate that exosomes can be modified *in vitro* and thereby provide them with added functions.

### Intact exosomes bound E-selectin under static and flow conditions in a Ca^2+^-dependent manner

**FIGURE 3:**
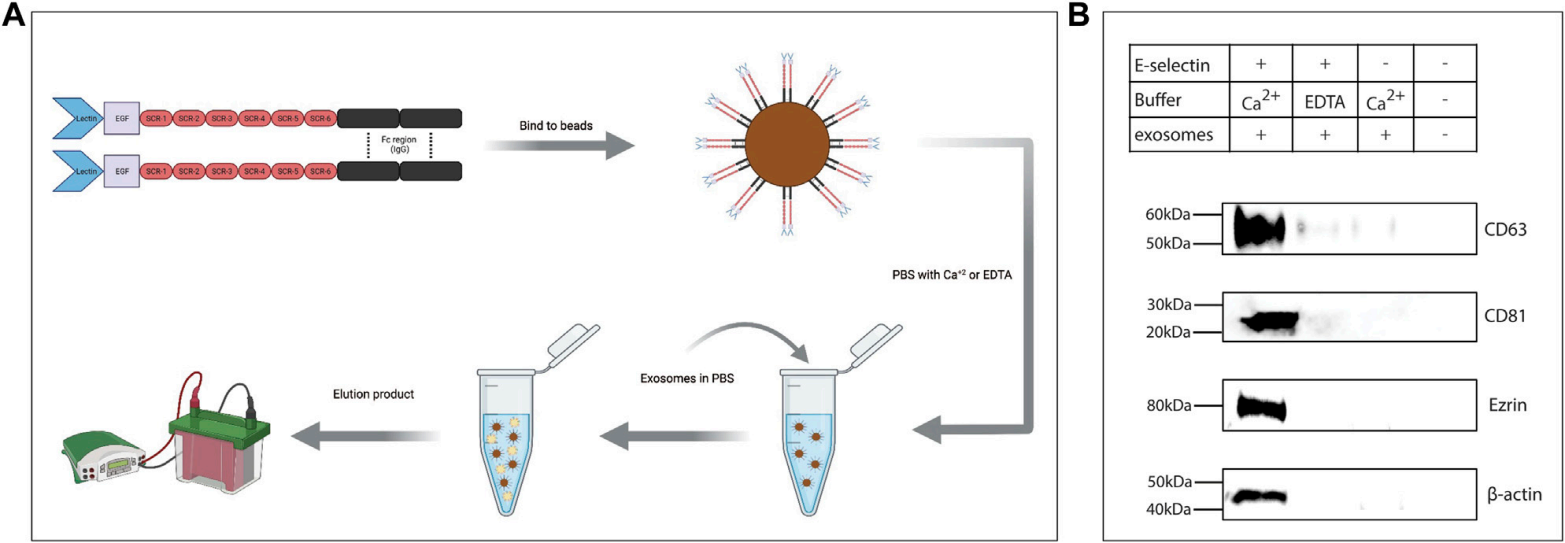
E-selectin is able to pull down intact exosomes expressing E-selectin ligands. **(A)** Cartoon illustrating the experimental workflow. rE-selectin-IgG was bound to magnetic beads coated with protein G. The complex was resuspended in PBS containing either 2 mM of Ca^2+^ or 20 mM of EDTA. Exosomes were added to that mixture, and after overnight incubation, the particles bound to the E-selectin-beads complex were eluted and lysates were analyzed by western blot. Refer to Materials and Methods for more details. Figure created with BioRender.com. **(B)** Western blot analysis of the eluted products from the E-selectin-bead complex. Control samples either containing EDTA to chelate out Ca^+2^ or beads (without the E-selectin) were used to verify the specificity of the interactions. A bead alone control (without exosomes) was also included. Blots were stained for the common markers of exosomes: CD63 and CD81, but also from proteins revealed from the Mass Spectrometry data such as ezrin and β-actin. This is representative of n = 3 independent experiments.

To determine if intact exosomes bound E-selectin, rE-selectin-IgG was used to immunoprecipitate whole exosomes derived from KG1a cells. Following an overnight incubation rotating at 4°C as illustrated in Figure 3A, the captured particles were eluted and prepared for western blot analysis. Blots were stained for surface exosomal proteins, CD81 and CD63, and internal proteins identified by the mass spectrometry analysis, ezrin and actin (“HSPCs-derived exosomes Mass Spectrometry raw data” in Datasheet 1). As shown in Figure 3B, the samples where Ca^2+^ was added to the immunoprecipitation showed clear signals for external and internal exosome proteins. As expected, samples where EDTA was added instead of Ca^2+^ or where protein G beads alone were used during immunoprecipitation of the exosomes (i.e., without the rE-selectin-IgG), no exosome markers were detected. A weak signal for CD63 was detected in the controls, that is, likely due to binding of the protein G beads to the immunoglobulin molecules of exosomes (“HSPCs-derived exosomes Mass Spectrometry raw data” in Datasheet 1). The same methodology was used to perform experiments with the K562-derived exosomes. When FTVI-treated and untreated K562-derived exosomes were incubated with the beads-E-selectin complex in the presence of calcium, only the FTVI-treated exosomes were able to bind to E-selectin (Supplementary Figure S5E). These data strongly suggest that intact exosomes bind to E-selectin in a Ca^2+^-dependent and sLe^x^–dependent manner.

To further support the validity of our findings, exosomes derived from primary CD34^+^ cells isolated from mobilized peripheral blood of healthy or AML donors were used. After examining the purity of these exosomes (Supplementary Figures S6A, B), total exosome lysates revealed several bands that bound E-selectin when blotted with rE-selectin-IgG (Supplementary Figures S6C, D). Moreover, using the experimental flow described in Figure 3A, the bead-E-selectin complex bound CD34^+^ primary cell derived exosomes in the presence of calcium and not in its absence (Supplementary Figures S6E, F). These data prove that intact exosomes derived from cell lines as well as primary cells have the ability to bind E-selectin.

**FIGURE 4:**
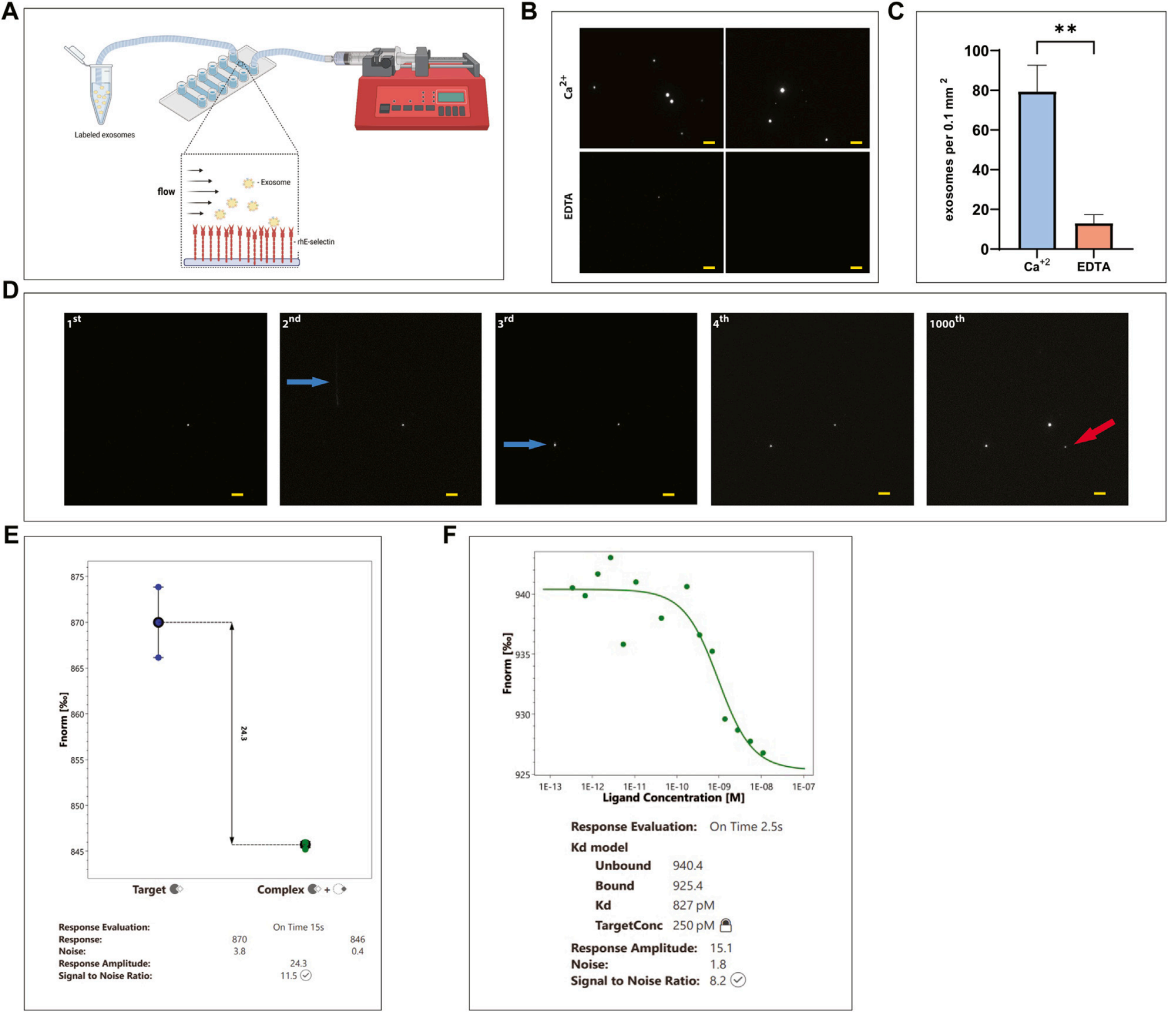
KG1a-derived exosomes bind E-selectin with high affinity and avidity even under flow. **(A)** Cartoon illustrating the experimental flow assay imaging setup showing labeled KG1a-derived exosomes flowing over rhE-selectin, that is, pre-deposited onto a microfluidics chamber. Figure created with BioRender.com. **(B, C)** DiD labeled KG1a-derived exosomes were introduced into a microfluidics chamber coated with rhE-selectin. Following 30 min of incubation, the chambers were washed and imaged using fluorescence microscopy. **(B)** In the chambers where Ca^+2^ was present (*upper* images), a plethora of exosomes were observed. However, in chambers where EDTA was present (*lower* images), exosomes were scarce. **(C)** The observed differences were quantified and expressed as number of exosomes observed per area, student’s t-test, *p*-value (**) < 0.01. **(D)** DiD labeled KG1a-derived exosomes were introduced into microfluidics chambers coated with rhE-selectin at a constant flow of 100 μl min^−1^ and increased up to 2000 μl min^−1^. Several frames were recorded to illustrate the binding of exosomes to the deposited E-selectin in real-time. In the 1st frame, one exosome is observed to be bound to the E-selectin. In the 2nd frame, another exosome entered the recording field (white line indicated by a blue arrow) bound firmly to the E-selectin by the 3rd frame (indicated by the blue arrow). This exosome remained bound stably to the E-selectin as shown in the 4th frame and even after 1000 frames (1000th frame) when the flow rate reached up to 2000 μl min^−1^. Furthermore, a new exosome bound at the recoding area (red arrow). Scale bar = 5 μm. **(E, F)** Microscale Thermophoresis (MST) assay between KG1a-derived exosomes and rE-selectin. **(E)** A *Binding Check* assay was performed using labeled rE-selectin and unlabeled KG1a-derived exosomes. As illustrated, a clear difference was apparent between the signal detected from the sample with the labeled E-selectin alone (Target, blue dots) and the sample with the labeled E-selectin mixed with the KG1a-derived exosomes (Complex, green dots), indicating binding detectable by the MST. **(F)** *Binding Affinity* assay performed using labeled rE-selectin and unlabeled KG1a-derived exosomes. The Kd was found to be in the level of pMolar, indicating a strong interaction between exosomes and rE-selectin. Results shown are representative of n = 3 independent experiments.

To image the binding of exosomes to E-selectin in a more direct way, a microfluidics chamber approach was used (Figure 4A). Exosomes were pre-stained with DiD dye and introduced to a microfluidics chamber where rhE-selectin was deposited on its surface. Exosomes were left to incubate with the surface of the coated chamber for 30 min without any shear force being introduced. After washing, the samples were observed under a fluorescence microscope to determine binding. Numerous fluorescently labeled exosomes were detected in the microfluidics chamber where Ca^2+^ was present (Figure 4B, *upper* panels), while in control samples where EDTA was present in the buffer instead of Ca^2+^, it was challenging to find a field where any exosomes bound to the E-selectin coated surface (Figure 4B, *lower* panels). Quantification of the bound exosomes (Figure 4C) confirms the Ca^2+^-dependent binding of the exosomes to E-selectin.

To observe the binding of exosomes to rhE-selectin coated microfluidics channels in real-time under physiological flow conditions, DiD-labeled KG1a-derived exosomes were introduced into the microfluidics chamber as in Figure 4A at flow rates ranging from 100–2000 μl min^−1^. Starting with a flow of 100 μl min^−1^, we observed exosomes “sticking” to the deposited E-selectin while passing throw the chamber (Figure 4D). This binding happened instantaneously and was firm. Even if the flow rate was increased up to 2000 μl min^−1^, the exosomes were not released from the surface of the chamber until the end of the experiment, indicating a very strong binding.

To further elaborate on the strength of binding of the exosomes to E-selectin, a Microscale Thermophoresis (MST) assay was developed to measure the binding affinity between E-selectin and the KG1a-derived exosomes. In preparation for the assay, the rE-selectin construct was labeled using the Monolith NT His-Tag Labeling Kit. After calculating the molarity of the exosomes (see Methods for details), we used the appropriate combinations of labelled E-selectin and exosomes, as recommended by the MO. Control v1.6.1 software (NanoTemper) for both “*Binding Check*” and “*Binding Affinity*” assay. As shown in Figure 4E, binding of labeled E-selectin was detected with KG1a-exosomes at concentrations of exosomes as low as 11 nM, indicating that the binding between exosomes and E-selectin is considerably strong. In fact, Kd measurements were found to be at the pMolar level, suggesting a strong interaction (Figure 4F), which is consistent with the flow data above.

### *In vivo* bio-distribution of exosomes is E-selectin dependent

**FIGURE 5:**
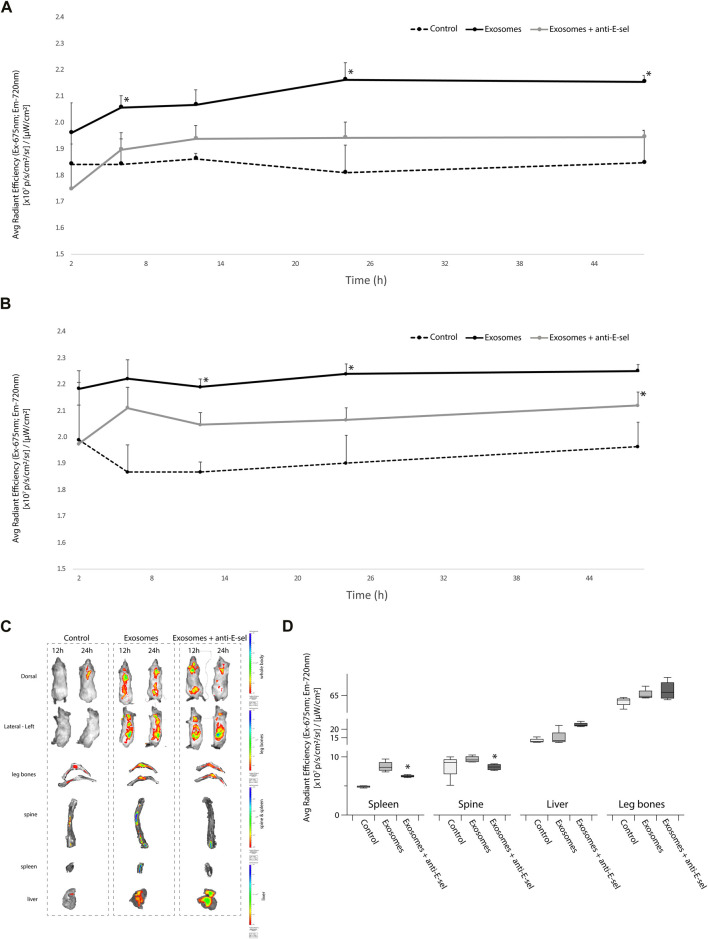
*In vivo* biodistribution of exosomes is influenced by E-selectin. KG1a-derived exosomes were prestained with VivoTrack 680 and delivered to NSG mice intravenously *via* the tail vein. NSG recipients were either pretreated with blocking anti-E-selectin antibody 3 h prior to exosome delivery (exosomes + anti-E-sel) or left untreated (exosomes). Control group mice that did not receive exosomes. **(A)** Quantitative analysis of fluorescence intensity, statistically analyzed by student’s t-test, using IVIS imaging of the whole body of NSG mice in the dorsal position at 2, 6, 12, 24, and 48 h is shown. The dorsal position is helpful to detect the fluorescence signal from the spine (*p*-value (*) <0.05, Exosomes group vs. Exosomes + anti-E-sel group). **(B)** Quantitative analysis of fluorescence intensity, statistically analyzed by student’s t-test, using IVIS imaging of the whole body of NSG mice in the left-lateral position at 2, 6, 12, 24, and 48 h is shown. The left-lateral position is helpful to detect the fluorescence signal from the spleen (*p*-value (*) <0.05, Exosomes group vs. Exosomes + anti-E-sel group). **(C)** Representative IVIS images of organs after 48 h of whole body IVIS imaging (at 12 and 24 h) following injection of VivoTrack 680-labeled KG1a-derived exosomes. Live IVIS imaging of mice from the dorsal (to show spine) and the lateral left (to show spleen) positions are shown at 12 and 24 h post-delivery of KG1a-derived exosomes or control group. Mice pretreated with anti-E-selectin blocking antibody 3 h prior to KG1a-derived exosome delivery are also shown. After imaging at 48 h, mice were sacrificed and organs were imaged *ex vivo* (spleen, liver, leg bones and spine). Representative images of these organs are shown. **(D)** Quantitative analysis of the fluorescence intensity of the leg bones, spine, spleen and liver are shown the exosomes accumulation in different organs and were statistically analyzed by student’s t -test (*p*-value (*) <0.05, Exosomes group *vs*. Exosomes + anti-E-sel group).

We next sought to determine the distribution of pre-stained KG1a-derived exosomes in a NSG mice that were either pretreated with blocking anti-E-selectin antibody or not. As shown in Figure 5A (dorsal view) and Figure 5B (lateral left view), IVIS imaging of the total body showed higher signals in the mice receiving KG1a-derived exosomes, which is significantly higher than in mice from the control group. Interestingly, when mice were pretreated with anti-E-selectin blocking antibody, the signal was significantly reduced. Moreover, images of the mice indicated that the majority of the accumulation of the signal centered around the spine (dorsal view) and the spleen and liver (lateral-left view) (Figure 5C, whole body images). Following the whole body IVIS imaging, mice were sacrificed, and the organs were further analyzed at 48 h after the delivery of exosomes. Indeed, as illustrated in Figure 5D, there was an accumulation of signal from exosomes in the spine, spleen, liver and leg bones. This signal was significantly reduced when E-selectin was blocked in the spleen and the spine. These data indicate that E-selectin is critical for the accumulation of the stained exosomes in the spine and spleen.

### Exosomes have the mechanisms to mediate changes in their cargo after their release

**FIGURE 6:**
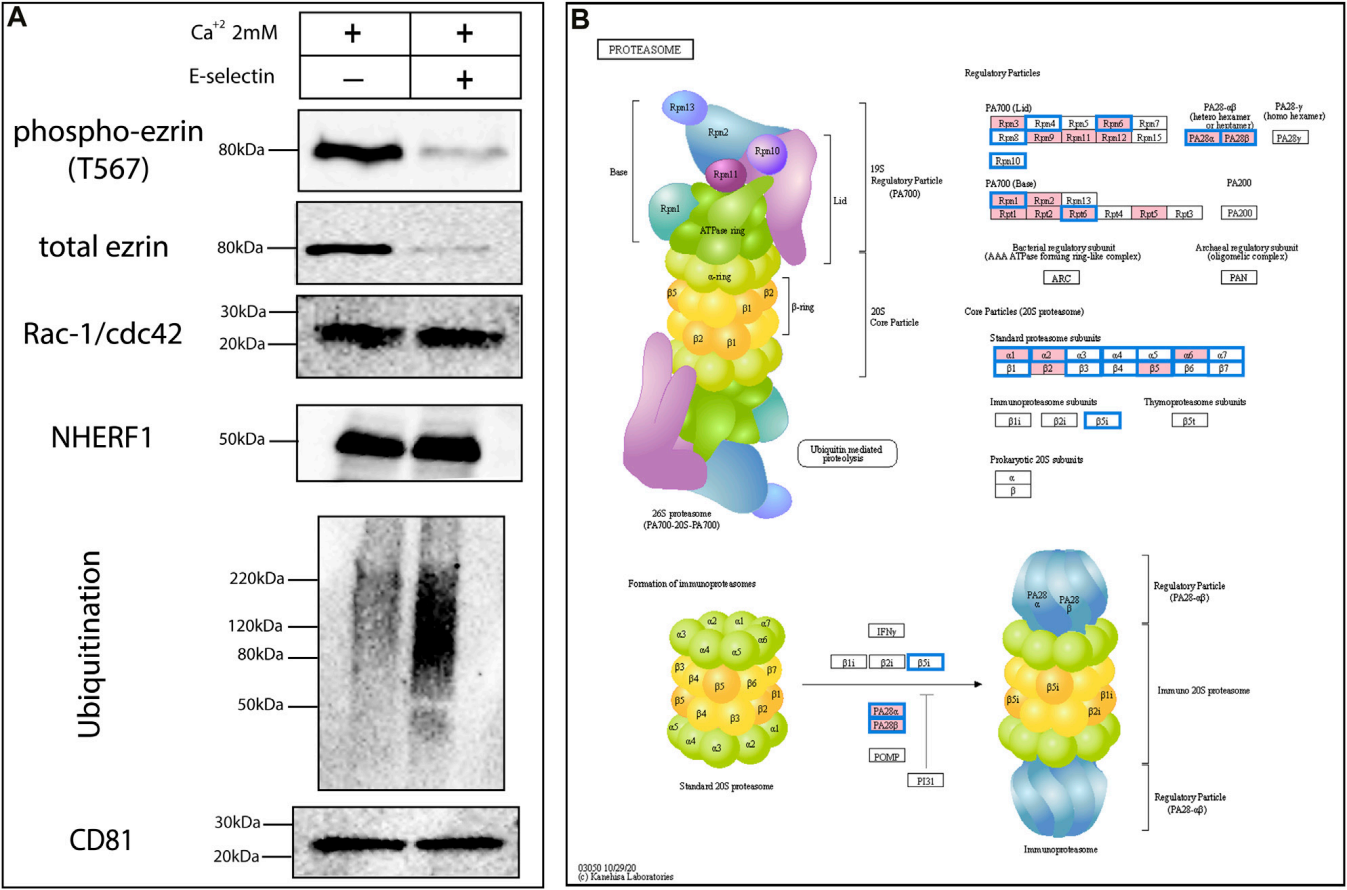
Exosomes may alter their cargo during transport to recipient cells in a non-stochastic manner. **(A)** Freshly derived KG1a exosomes were treated with E-selectin in the presence of Ca^2+^ to mediate binding. Subsequently, lysates were prepared and several signaling proteins were analyzed by western blot including ezrin, phospho-ezrin, Rac-1/Cdc42 and NHERF-1. Interestingly, there was an overall increase in ubiquitination observed in response to E-selectin binding but not all proteins analyzed were perturbed. CD81 blot was used as a loading control for the western blots illustrated. Results shown are representative of n = 3 independent experiments. **(B)** The proteomics analysis of the KG1a (pink) and healthy HSPCs (blue frame)-derived exosomes revealed the presence of proteins related to the proteosome indicating that exosomes contain the machinery to enable them towards controlled degradation of proteins.

A more in-depth analysis of the Mass Spectrometry data of the KG1a-derived exosomes revealed a plethora of signaling molecules which might be functional in exosomes as they are inside the cytoplasm of cells. Therefore, using E-selectin, we tried to trigger putative pathways that are related to E-selectin- ligand(s) interaction. A very good candidate to examine putative signaling-related alternations was ezrin due it is abundance in exosomes as well as its essential role in adhesion and migration processes, linking the cytoskeleton to the cell membrane (Gautreau et al., 1999). Ezrin has a distinct phosphorylation site at Thr567 which plays a crucial role in its interactions (Parnell et al., 2015). To test if signaling changes in ezrin occur within exosomes upon interacting with E-selectin, KG1a-derived exosomes were placed in PBS containing 2 mM Ca^2+^ and then treated with the rE-selectin for 1 h. As illustrated in Figure 6A, the amount of phosphorylated ezrin at Thr567 was drastically reduced when the exosomes were treated with E-selectin compared to exosomes treated with Ca^2+^ alone. However, the amount of the total ezrin was also reduced, suggesting that the reduction in phosphorylation was likely due to degradation of ezrin and not due to dephosphorylation. Examination of two other proteins, Rac-1 and NHERF1, that were identified at the proteomics data did not follow the reduction of total ezrin. In contrast, the total amount of Rac-1 and NHERF1 proteins were equal after E-selectin treatment in KG1a-derived exosomes (Figure 6A). Therefore, a mechanism which targets specific protein(s) for degradation seemed to be activated and not just a general mechanism aiming to stochastically degrade proteins (i.e., caspases). Indeed, it may involve a process of ubiquitination as the total polyubiquitination was increased after E-selectin treatment. Furthermore, based on the proteomics data, the HSPCs-derived exosomes do possess proteins involved in ubiquitination (see file in Datasheet 3: “Ubiquitin protein ligase binding protein list identified in HSPCs-derived exosomes”), but also contain the proteosome proteins (Figure 6B), as illustrated from the analysis of the proteomics data.

## Discussion

Since their discovery, exosomes have been linked to many biological functions such as cell-to-cell communication and cell reprogramming among others (Balaj et al., 2011; Hong et al., 2017; Ringuette Goulet et al., 2018). For the exosomes to cause alterations in the recipient cell, they need to interact with it, either releasing their cargo inside the cells by merging with the cell membrane or activating the cell’s ligands *via* adhesion (Mathieu et al., 2019). Studying the proteome of the exosomes can give indications of their potential functions. The proteomics analysis of exosomes derived from different blood cells (Diaz-Varela et al., 2018) as well as our analysis of AML derived exosomes show that exosomes contain adhesion-related proteins that can possibly interact with other adhesion molecules. In particular, we uncovered several proteins involved in the migration of numerous cell types such as selectin ligands, chemokines and integrins (Supplementary Table S3) suggesting that similar adhesion and migration functions could take place in exosomes as in their parental cells (Figure 1C). Here using as a model, the CD34^+^ AML cell line KG1a, a cell line previously described to possess optimum cell migration characteristics both *in vitro* and *in vivo* (Dimitroff et al., 2001; Merzaban et al., 2011; AbuSamra et al., 2015; AbuSamra et al., 2017; Ali et al., 2017; AbuZineh et al., 2018; AbuElela et al., 2020; Aleisa et al., 2020; Al Alwan et al., 2021), we investigated the dynamic potential of KG1a-derived exosomes to interact with E-selectin. Our data were further confirmed using exosomes derived from healthy and AML primary CD34^+^ HSPCs cells. To our knowledge, this is the first study illustrating the functional interaction and characterization of exosomes with E-selectin, a key adhesion molecule constitutively expressed by the endothelial cells of the hematopoietic tissues (Schweitzer et al., 1996).

In this study, the interaction of E-selectin with exosomal ligands appeared to mimic that of the interaction between E-selectin and its cellular ligands. E-selectin bound to its exosomal E-selectin ligands in a Ca^2+^-dependent manner that relied on sLe^x^ glycosylation of the ligands. However, in contrast to CD34, CD44, and PSGL-1, exosomal CD43 did not bind to E-selectin although its parent cell, KG1a (Merzaban et al., 2011), does. This is interesting and is likely due to the fact that the exosomal CD43 does not contain the sLe^x^ decorated form, that is, recognized by E-selectin. This study further reports the specific Ca^2+^-dependent and sLe^x^-dependent binding of whole, intact exosomes to E-selectin molecules (Figure 3). This approach can be also used as a method to separate exosomes that express E-selectin ligands from a mixture of exosomes. Moreover, real-time imaging of KG1a-derived exosomes flowing over immobilized E-selectin confirmed the Ca^2+^-dependent nature of this interaction under various flow rates. Fluorescently stained exosomes were initially brought into the E-selectin coated microfluidics chamber at a flow rate of 100 μL min^-^1 and were able to bind strongly and firmly to E-selectin under constant flow (Figure 4D). The interactions that were achieved between the exosomes and the E-selectin were maintained even at flow rates upwards to 2000 μl min^−1^ and did not detach. These data illustrate strong binding of exosomes to E-selectin. Using an MST approach, quantification revealed that the Kd of this interaction was indeed found to be in the pMolar range (Figure 4F). Previously published work using the SPR technique, quantified the binding of E-selectin to different E-selectin ligands isolated from KG1a cell lysates, and reported Kd values in the hundreds of nMolar (AbuSamra et al., 2017; Aleisa et al., 2020). However, as recently shown, when proteins are on the surface of a lipid vesicle, they bind stronger to their ligands (Zhou et al., 2021), which may explain why the Kd of the exosomes-E-selectin interaction is significantly lower compared to the previously mentioned work.

Exosomes derived from blood malignancies can alter the profile of endothelial cells (Wang et al., 2019). Guided delivery of exosomes to endothelial cells could be achieved through their *in vitro* modification. For example, *in vitro* modification of exosomes using the fucosyltrasferase VI enzyme (Al-Amoodi et al., 2020), created many epitopes on membrane proteins that could function as E-selectin ligands. Indeed, exosomes derived from K562 cells, a cell line that does not bind to E-selectin, could be manipulated to bind E-selectin following *in vitro* treatment with the FTVI enzyme. Therefore, exosomes can undergo dynamic changes and be given new properties. Given that exosomes can be used as a drug delivery system (Tang et al., 2012; Tian et al., 2014), these findings can assist in increasing the specificity of drug delivery to particular cells or tissues, i.e., where E-selectin is expressed such as under inflammatory conditions.

As indicated by the *in vivo* study documented here, E-selectin has a crucial role in the targeting of AML exosomes to specific organs such as the spleen (Figures 5B–D). Many studies have described AML cell engraftment to the spleen as well as the bone marrow in xenotransplantation studies (Her et al., 2017). In patients with acute myeloid leukemia and other leukemias, enlarged spleens are associated with poor survival after treatments involving allogeneic stem cell transplantation (Shimomura et al., 2018; Yuasa et al., 2020). Interestingly, leukemic cells have been described to migrate to as well as proliferate more in the spleen more than the bone marrow (Ma et al., 2014). Moreover, the spleen has been described as a “sanctuary” where leukemic cells can thrive outside of the bone marrow in order to escape from drug therapies (Di Grande et al., 2021) suggesting the importance of developing drug delivery systems targeting this organ. Better understanding of the splenic tumor microenvironment and the role of exosomes in the recruitment and maintenance of leukemic cells is invaluable knowledge needed in the treatment of leukemia and other diseases.

Another position that showed E-selectin dependent exosome accumulation was the spine. Patients with AML can present with central nervous system involvement in rare cases (∼3% of patients) (Landis and Aboulafia, 2003; Shihadeh et al., 2012) likely due to leukemic cell invasion (Asano et al., 2016). Here we show that exosomes may be important players in mediating this cell invasion due to their accumulation in the spinal bone marrow. These data suggest organotropic distribution of exosomes based on their ability to bind to E-selectin. Previous studies focused on integrins, adhesion molecules involved in cell-cell and cell-matrix interactions, illustrated that specific integrin expression guided exosomes to particular organs that became the site of future metastases (Hoshino et al., 2015). Therefore, disrupting such organ-specific infiltration of exosomes could alter the migration and metastasis of cancer cells.

Trying to understand the biological need underlying the ability of AML CD34^+^cells-derived exosomes to bind to E-selectin- ergo targeting endothelial cells-the answer can be found in the cargo of these exosomes that will be delivered to their target cells. Apart from the protein cargo, which was mentioned before, by analyzing the miRNAs found in KG1a-derived exosomes (Xu et al., 2020), several crucial targets were identified (Supplementary Figure S7). The PI3K-AKT pathway was found to have the most targets among the other KEGG pathways (see “KG1a-derived exosomes-miRNA target analysis” in Datasheet 4). Indeed, PTEN, the main negative regulator of this pathway was a target of at least 15 exosomal miRNAs. Experimental data have demonstrated that miR-26a, one of the higher in abundance in KG1a-derived exosomes, has been found to directly down-regulate PTEN (Coronel-Hernandez et al., 2019), and has also been shown to provide anti-apoptotic properties (Zhang et al., 2015). Additionally, activation of the PI3K-AKT pathway can lead to angiogenesis of endothelial cells (Karar and Maity, 2011). Moreover, another pathway controlling angiogenesis, Wnt (Dejana, 2010), was also a top target of these exosomal miRNAs. Interestingly, the Extracellular Matrix (ECM) receptor interaction category was a major target of KG1a exosomal miRNAs suggesting that they could facilitate the remodeling of the microenvironment at a particular location, such as the bone marrow. For instance, exosomal miRNAs target five members of the laminin family, a main component of ECM (Siler et al., 2000). One of them, laminin 4, was found to be targeted by at least four exosomal miRNAs, including miR148a, the third most abundant miRNA in KG1a-derived exosomes. In addition to this, recent studies have shown that depletion of laminin 4 from the bone marrow can lead to loss of normal hematopoiesis but acceleration of AML (Cai et al., 2022).

Although exosomes are mainly considered as cargo transporters of active biomolecules, these transporters could ultimately undergo dynamic changes during the transportation process. For instance, the reported binding of the exosomes to E-selectin could merely deliver the exosome cargo but binding could also lead to further functional changes to the exosomes themselves. The proteomics data presented here, illustrates that exosomes contain a plethora of signaling molecules, which could be functional in both exosomal and cellular environments they interact with. Indeed, by treating the KG1a-derived exosomes with E-selectin, we observed changes inside the exosomes themselves. This observation is intriguing as it indicates that the exosomal cargo could undergo alterations (i.e., phosphorylation/dephosphorylation, ubiquitination) after its release from the parental cell until its final uptake by the recipient cell. One could envision that depending on the receptor ligand interaction, different sets of signaling pathways could be set off resulting into modification of the exosomal cargo on its way to the recipient cell. These data also imply that exosomes should be considered as part of a system and not only independently, as exosomes seem to modify their cargo according to the recipient cells that they interact with.

## Data Availability

The original contributions presented in the study are included in the article/Supplementary Material, further inquiries can be directed to the corresponding author.
