# Supplementary material for: CD34+ HSPCs-derived exosomes contain dynamic cargo and promote their migration through functional binding with the homing receptor E-selectin
Source: Front Cell Dev Biol. 2023 Apr 25;11:1149912. doi: 10.3389/fcell.2023.1149912 (PMC10166801; doi:10.3389/fcell.2023.1149912)
Supplement: Supplementary file 1 [file DataSheet1.zip › Supplementary Materials.pdf]

Supplementary Material

CD34$^+$ HSPCs-derived exosomes contain dynamic cargo and promote their migration through functional binding with the homing receptor E-selectin

Ioannis Isaioglou[1], Mansour Aldehaiman[†1], Yanyan Li[†1], Abdellatif Ait Lahcen[⊕2], Sakandar Rauf[⊕2], Asma S. Al-Amoodi[1], Umme Habiba[1], Abdullah Alghamdi[1], Shuho Nozue[1], Satoshi Habuchi[1], Khaled N. Salama[2], Jasmeen S. Merzaban[1*]

*** Correspondence:** Jasmeen Merzaban: jasmeen.merzaban@kaust.edu.sa

1. Bioscience Program, Biological and Environmental Science and Engineering Division, King Abdullah University of Science and Technology (KAUST), Thuwal 23955-6900, Kingdom of Saudi Arabia

2. Electrical and Computer Engineering Program, Computer, Electrical and Mathematical Science and Engineering Division, King Abdullah University of Science and Technology (KAUST), Thuwal 23955-6900, Kingdom of Saudi Arabia

†: These authors contributed equally

⊕: These authors contributed equally

Supplementary Tables

Supplementary Table 1. Antibodies used for immunoprecipitation assays. For each experiment 3µg of each antibody was used.

Antibody	Vendor	Catalog number
Anti-CD34	Santa Cruz Biotechnology	sc-19587
Anti-CD43	R&D Systems	MAB2038
Anti-CD44	BD Biosciences	550990
Anti- CD162 (PSGL-1)	BioLegend	328802

Supplementary Table 2. Antibodies used for western blots in this study. All the primary antibodies were diluted in 1 x TBST and were used at a concentration of ~1µg/mL except the antibody for the β-actin* which was used at a concentration of 0.2µg/mL. The secondary antibodies were used in a concentration of 0.1µg/mL diluted in 1 x TBST.

Antibody	Vendor	Catalog number
Anti- CD81	Abcam	ab79559
Anti- TSG101	Abcam	ab83
Anti- CD44	Abgent	AM1901b
Anti- CD43	Thermo Fisher	MA5-16339
Anti- CD34	Bio-Rad	MCA547G
Anti- CD162 (PSGL-1)	BioLegend	328802
Anti- CD63	Thermo Fisher	Ts63
Anti- Ezrin	Cell Signaling	3142
Anti-Phospho-Ezrin(Thr567)	Thermo Fisher	PA5-37763
Anti- β-actin*	Thermo Fisher	AM4302
Anti-Human Cutaneous Lymphocyte Antigen (HECA)	BD Bioscience	555946
Anti- Rac1/Cdc42	Cell Signaling	4651
Anti-Uubiquitinylation	Enzo Life Sciences	ENZ-ABS840
Anti-EBP50(NHERF1)	Thermo Fisher	PA1-090
Anti-mouse IgG, HRP	Cell Signaling	7076
Anti- rabbit IgG, HRP	Cell Signaling	7074
Anti-rat IgM, HRP	Bio-Rad	302005
Anti-human IgG-Fc, HRP	Merck	AP1113P

Supplementary Table 3. List of integrins identified in the lysates of the respective exosomes.

Gene name	Uniprot ID	KG1a	Healthy HSPCs
ITGAL	P20701, H3BSV1, B4E021	+	+
ITGB1	P05556, C9JPK5, E7ERX5	+	+
ITGB1, isoform 2	P05556-2	+	+
ITGB2	A0A494C0X7, J3KNI6, E7EVZ9, D3DSM0, P05107	+	+
ITGB3	P05106	−	+
ITGB4	J3QRK0	−	+
ITGB5	H7C580	−	+
ITGB7	F5H6T4	−	+
ITGA4	E7EP60, P13612	+	+
ITGA5	P08648	+	+
ITGA6	P23229	+	−
ITGA6, isoform 9	P23229-9	−	+
ITGA2B	A0A3B3IU79	+	−
ILK (integrin-linked protein kinase)	A0A0A0MTH3	+	−

Supplementary Figures:

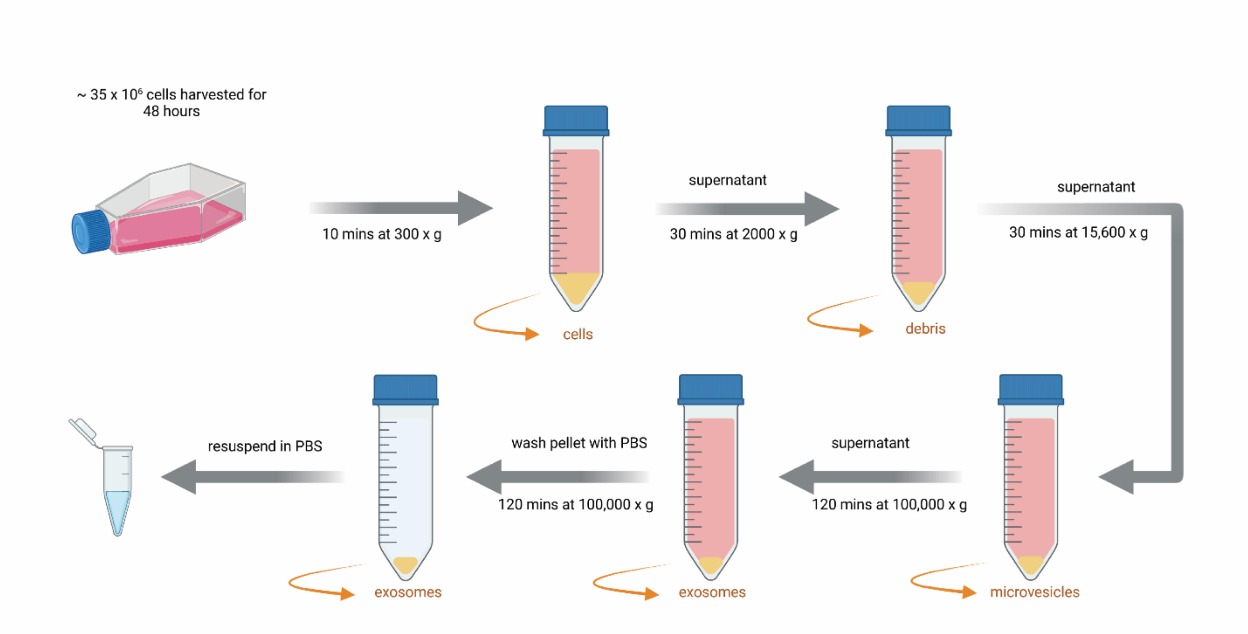

Figure S1: Exosome isolation process. Cartoon illustrating the isolation protocol of exosomes derived from cell types used throughout the manuscript. Following a starvation of the cells for 48 hours, serial centrifugation steps took place to remove larger particles from the media. By the last ultracentrifugation step, exosomes were pelleted and finally resuspended in PBS buffer. Figure created with BioRender.com.

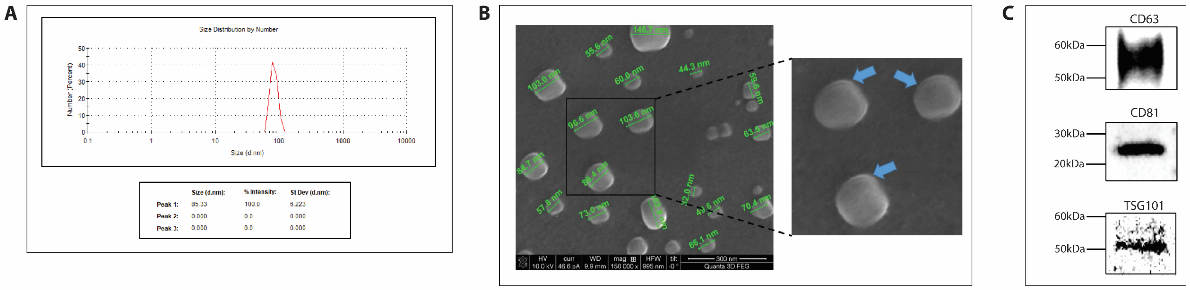

Figure S2: Characterization and quality control of cell derived exosome preparations. Characterization of KG1a-derived exosomes was performed in numerous assays. **(A)** Dynamic Light Scattering (DLS) data illustrated that the average diameter of the isolated exosomes was 85 nm, without detection of other particles larger or smaller than the expected diameter. **(B)** Scanning Electronic Microscopy images demonstrated the size and the shape of the isolated particles. The exosomes had a spherical shape, with their diameter varying between 40nm-150nm. A lipid membrane bilayer was noticeable (blue arrows). **(C)** Western-blot analysis of the isolated KG1a-derived particles revealed the existence of common exosomal markers such as CD63, CD81 (transmembrane markers), and TSG101 (internal exosomal marker) at their respective molecular weights.

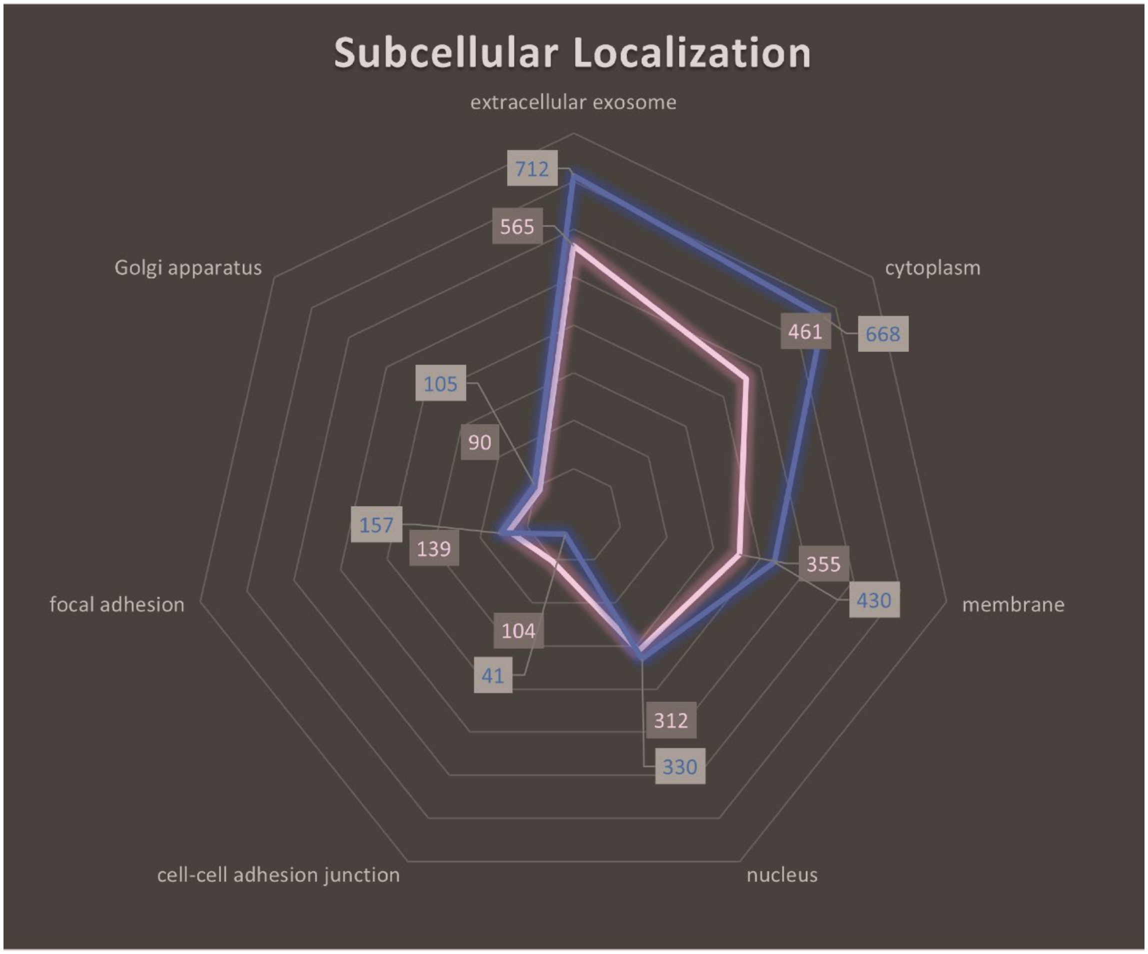

Figure S3: Subcellular localization analysis of HSPCs-derived exosomal proteins. Radar diagram illustrating the top seven subcellular localization candidates of the exosomal proteome (KG1a-pink, healthy HSPCs-blue). The closer to the edge of the diagram, the more proteins are related to the particular subcellular component referenced. The "extracellular exosome" category generated the highest hit confirming the successful isolation of exosomes.

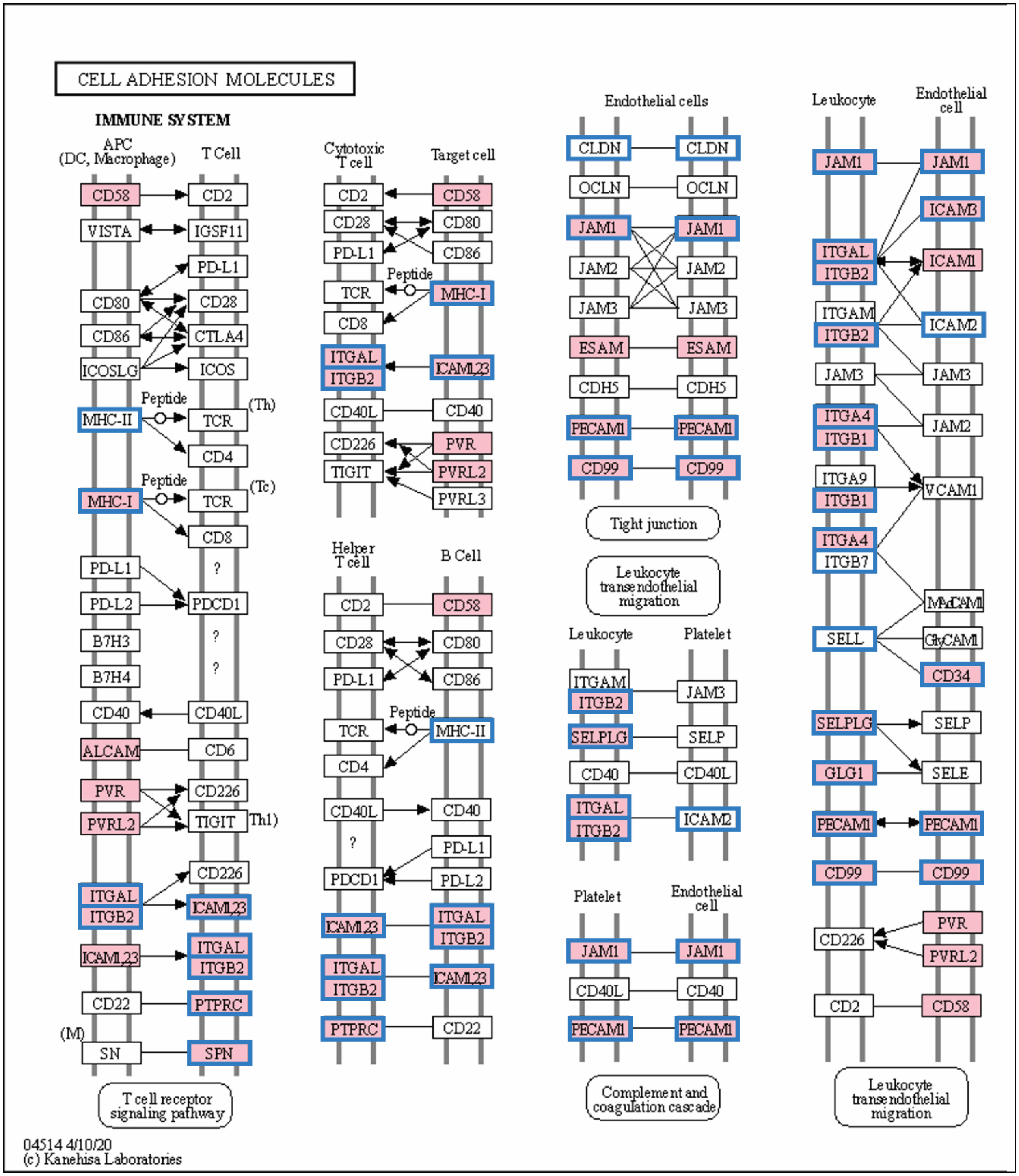

Figure S4: Cell Adhesion Molecule KEGG Map (modified). The proteomics analysis of the KG1a (pink) and healthy HSPCs (blue frame)-derived exosomes revealed proteins related to this pathway. Among them, integrins, junction adhesion molecules, and cellular receptors such as CD34, PTPRC, and CD99 were identified.

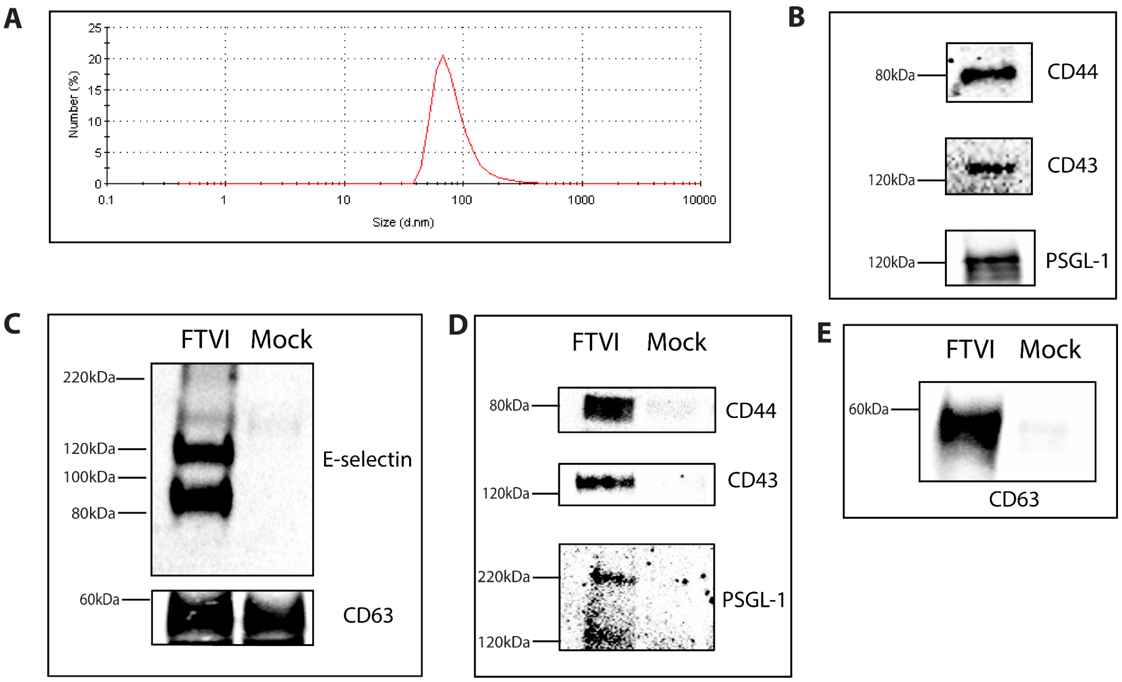

Figure S5: Fucosylation of K562-derived exosomes promotes E-selectin binding. (A) Dynamic Light Scattering (DLS) data illustrated that the average diameter of the isolated exosomes was 84 nm, without detection of other particles larger or smaller than the expected diameter. **(B)** Western blot analysis of lysates of K562-derived exosomes revealed the presence of CD43, CD44 and PSGL-1 at the expected molecular weights. **(C)** Lysates were prepared from K562-derived exosomes that were treated with FTVI or buffer-treated (Mock) and subjected to Western blot analysis to detect binding to E-selectin (rE-selectin-IgG). Note that only in lysates derived from exosomes treated with FTVI were several potential E-selectin ligands revealed, while no clear bands were detected at the lysates derived from the control exosomes (Mock). **(D)** rE-selectin-IgG was used to immunoprecipitate proteins from lysates of FTVI-treated or buffer-treated K562-derived exosomes in the presence of 2mM of Ca^{2+}. Western blot analysis revealed the presence of CD43, CD44, and PSGL-1 in the samples derived from the FTVI-treated exosomes but not from samples derived from buffer-treated exosomes confirming the selective, sLe^x-dependent binding of E-selectin to its ligands. **(E)** The methodology of this experiment is illustrated in Figure 3a. Intact K562-derived exosomes were incubated with beads-E-selectin complex. Subsequently, the exosomes eluted from the beads were blotted against CD63, a common exosomal marker. Only the exosomes treated with the FTVI prior to the incubation bound to the complex (FTVI) while the buffer-treated exosomes (Mock) did not. Results shown are representative of n = 3 independent experiments.

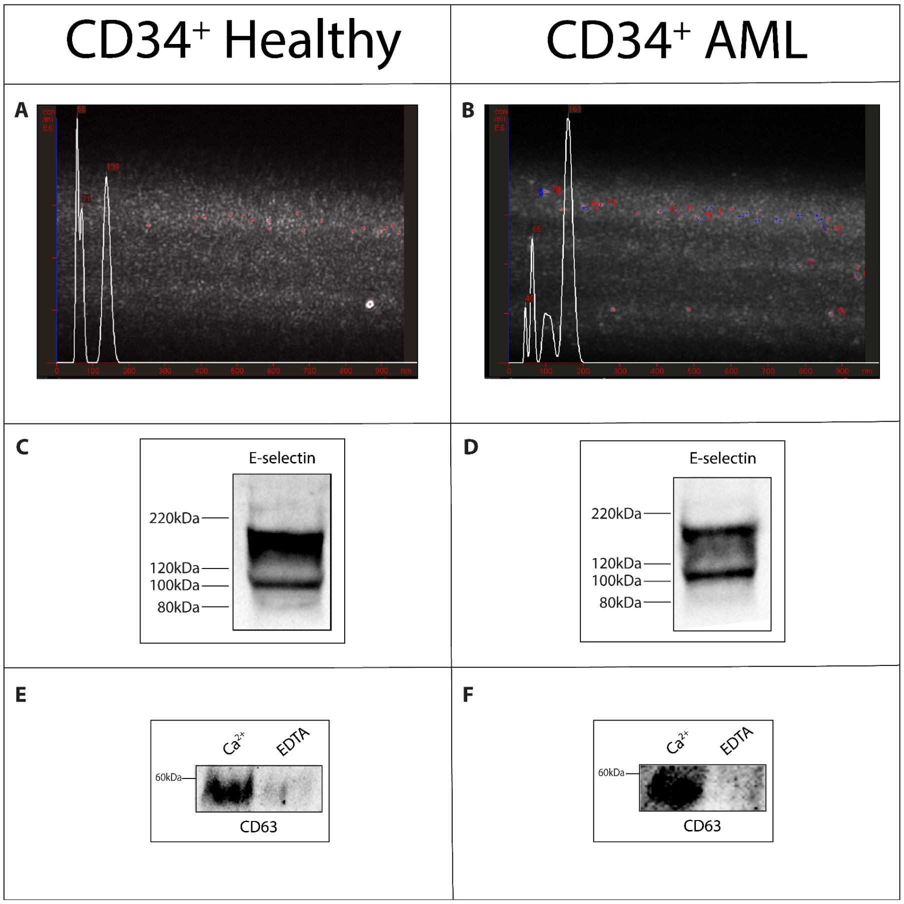

Figure S6: Exosomes derived from primary CD34$^+$ cells interact with E-selectin. (A-B) Size mesaurements of exosomes derived from GCSF-mobilized CD34$^+$ peripheral blood isolated from healthy donors **(A)** or patients with AML disease **(B)** using the NTA system. Exosomes exhibited a diameter within the expected range without the presence of larger particles. **(C-D)** Western blot analysis of lysates derived from GCSF-mobilized CD34$^+$ peripheral blood isolated from healthy donors **(C)** or patients with AML disease **(D)** blotted with rE-selectin-IgG. **(E-F)** The methodology of this experiments is illustrated in Figure 3a. Intact exosomes derived from GCSF-mobilized CD34$^+$ peripheral blood isolated from healthy donors **(E)** or patients with AML disease **(F)** were incubated with beads-E-selectin complex in the presence (Ca^{2+}) or absence (EDTA) of 2mM Ca^{2+}. The elution products were blotted against CD63, a common exosomal marker.

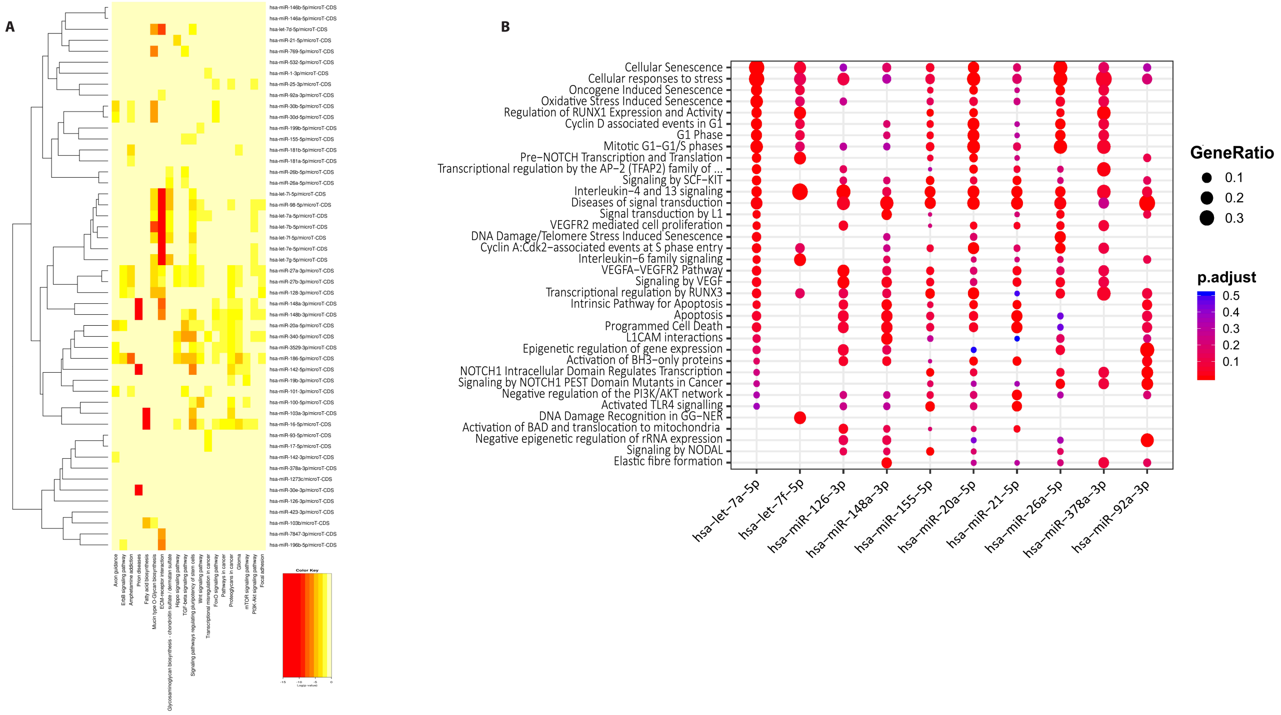

Figure S7: Target analysis of KG1a-derived exosomal miRNAs. (A) Heat map of KEGG pathways targeted by KG1a-exosomal miRNAs. Exosomal miRNAs seemed to target in clusters specific pathways. Figure was created by the DIANA online tool. **(B)** Reactome dotplot representing the targeting of the most highly expressed miRNA to the respective Reactome category. The size of the dot represents the gene ratio and the color of the dots the adjusted-p-value (p-adjust). Figure was created by the MIENTURNET online tool.

Supplementary Files

Datasheet 1. "**HSPCs-derived exosomes Mass Spectrometry raw data**": Excel file including the raw Mass Spectrometry data as they were extracted by the MASCOT software aligned to the Uniprot Human database.

Datasheet 2. "**HSPCs-derived exosomes proteome analysis**": Excel file containing the KEGG pathway, and the Gene Ontology-Biological Process enrichment analysis of the proteins identified in the leukemic and healthy HSPCs-derived exosomes. Each sheet includes the pathways enriched, the total number of proteins found to be involved in each pathway, as well as the p-value and the Benjamini value of the enrichment of each pathway.

Datasheet 3. "**Ubiquitin protein ligase binding protein list identified in HSPCs-derived exosomes**": Excel file including the proteins identified in the category “ubiquitin protein ligase binding” by the DAVID online tool, using GO-Molecular Function analysis both for leukemic and healthy HSPCs-derived exosomes. For each protein, the Uniprot ID, Description, and Symbol are provided.

Datasheet 4. "**KG1a-derived exosomes-miRNA target analysis**": Excel file including five sheets; The first one contains the top 50 miRNA ID and their transcripts per million identified in KG1a-derived exosomes and used for our analysis. The second sheet has the list of targets by these miRNAs, including p-values and the number of interactions per target (Mienturnet software). The third sheet has the KEGG pathways found to be targeted, providing information about the p-value, the number of targets per pathway, and the number of miRNAs that target each pathway (DIANA software). The fourth sheet has also the same analysis but for the REACTOME pathways (Mienturnet software). The last sheet contains the miRNAs that can target laminin 4 (LAMA4). Among them, four (green highlighted) were found to be at the top 50 miRNAs in KG1a-derived exosomes.
